# Characterization of tumor microenvironment and programmed death-related genes to identify molecular subtypes and drug resistance in pancreatic cancer

**DOI:** 10.3389/fphar.2023.1146280

**Published:** 2023-03-17

**Authors:** Liang Yu, Risheng He, Yunfu Cui

**Affiliations:** ^1^ Department of Hepatopancreatobiliary Surgery, Second Affiliated Hospital of Harbin Medical University, Harbin, China; ^2^ Department of Pathology, School of Clinical Medicine, Li Ka Shing Faculty of Medicine, The University of Hong Kong, Hong Kong, China

**Keywords:** PAAD, tumor microenvironment, immunotherapy, methylation, molecular subtypes, prognosis, programmed cell death, drug resistance

## Abstract

**Background:** Immunotherapy has been a key option for the treatment of many types of cancer. A positive response to immunotherapy is heavily dependent on tumor microenvironment (TME) interaction. However, in pancreatic adenocarcinoma (PAAD), the association between TME mode of action and immune cell infiltration and immunotherapy, clinical outcome remained unknown.

**Methods:** We systematically evaluated 29 TME genes in PAAD signature. Molecular subtypes of distinct TME signatures in PAAD were characterized by consensus clustering. After this, we comprehensively analyzed their clinical features, prognosis, and immunotherapy/chemotherapy response using correlation analysis, Kaplan-Meier curves analysis, ssGSEA analysis. 12 programmed cell death (PCD) patterns were acquired from previous study. Differentially expressed genes (DEGs) were acquired based on differential analysis. Key genes affecting overall survival (OS) of PAAD were screened by COX regression analysis and used to develop a RiskScore evaluation model. Finally, we assessed the value of RiskScore in predicting prognosis and treatment response in PAAD.

**Results:** We identified 3 patterns of TME-associated molecular subtypes (C1, C2, C3), and observed that clinicopathological characteristics, prognosis, pathway features and immune features, immunotherapy/chemosensitivity of patients were correlated with the TME related subtypes. C1 subtype was more sensitive to the four chemotherapeutic drugs. PCD patterns were more likely to occur at C2 or C3. At the same time, we also detected 6 key genes that could affect the prognosis of PAAD, and 5 genes expressions were closely associated to methylation level. Low-risk patients with high immunocompetence had favorable prognostic results and high immunotherapy benefit. Patients in the high-risk group were more sensitive to chemotherapeutic drugs. RiskScore related to TME was an independent prognostic factor for PAAD.

**Conclusion:** Collectively, we identified a prognostic signature of TME in PAAD patients, which could help elucidate the specific mechanism of action of TME in tumors and help to explore more effective immunotherapy strategies.

## 1 Introduction

Pancreatic adenocarcinoma (PAAD), which is a malignant tumor developed from follicular cells and pancreatic duct epithelial cells, is also the most frequent malignancy in the digestive system (Bengtsson et al., 2020). PAAD patients are often diagnosed at locally advanced or metastatic stage due to a lack of early clinical symptoms at early stage, therefore missing the option of surgical treatment (Skelton et al., 2017). Although breakthroughs have been made in targeted molecular therapy, and immunotherapy, comprehensive therapy, PAAD prognosis is still unsatisfactory, showing an average survival for 5 years of lower than 5% due to late diagnosis, drug resistance, and recurrence, postoperative metastasis (Siegel et al., 2019). Moreover, Blockade of immune checkpoints by anti-CTLA-4 and/or anti-PD-1/anti-PD-L1 agents leads to T cell activation and provides an effective approach for tumor immunotherapy (Schizas et al., 2020). However, despite demonstrating robust results in certain malignancies, most phase I and II clinical trials have failed to show any clinical efficacy in PAAD (Bear et al., 2020; Carpenter et al., 2021). With the rapid development of biotechnology, some tumor molecular markers are increasingly playing an active role in the field of cancer treatment. Developing personalized precision medicine based on these biomarkers may help prolong survival time of cancer patients. Therefore, identifying prognostic biomarker to accurately estimate the survival outcome of those with PAAD has become an urgent task.

With the in-depth study of immunology, more and more evidences have shown that in the tumor microenvironment (TME), various immune cells play key roles in the occurrence and development of tumors (Hinshaw and Shevde, 2019). Interaction of tumor cells with tumor-infiltrating immune cells, immune response and cell transformation in TME may inhibit tumor development or promote its occurrence and also significantly affect the treatment response and clinical prognosis of patients (Xiao and Yu, 2021). At present, some studies have found that immune cells can be used as reliable and stable prognostic indicators for tumor patients. For example, Zuo and colleagues et al. demonstrated that high immune cell infiltration is inversely associated with the prognosis of glioblastoma multiforme, and uveal melanoma, low-grade glioma (Zuo et al., 2020). The above findings suggested that immune cell infiltration affect the prognosis of different cancers differently. However, there are few studies on the immune infiltration of TME in PAAD, and the specific molecular mechanism is still unclear. Systematic and in-depth research on TME of PAAD is expected to provide new insights for clinical treatment.

For a variety of tumors, immunotherapy is an emerging treatment modality and multiple clinical trials have demonstrated its efficacy in cancer treatment (Wang et al., 2019), (Chalabi et al., 2020), (Sheih et al., 2020). For example, CD8 T cells may be the predictive marker of immunotherapy response (St Paul and Ohashi, 2020). Accumulating evidence indicates that TME is a major contributor to tumor aggressive behavior and affects tumor patient response to immunotherapy (Bader et al., 2020). Immunotherapy provides more options for cancer treatment. However, recent clinical trials have shown that patients with PAAD are less likely to benefit from immunotherapy and some would experience some adverse events such as lymphopenia (Xie et al., 2020), (O'Reilly et al., 2019). Hence, clarifying the specific mechanism of action of TME in PAAD to divide patients with different characteristics into different molecular subtypes and predict their immunotherapy effects is expected to be a promising strategy.

In this study, applying sequencing data from the PAAD project in the ICGC and TCGA databases, we identified 3 molecular subtypes in PAAD according to the TME-related genes and systematically analyzed the prognosis of TME in different states, chemotherapy, and immunotherapy response. Next, we screened characteristic genes related to PAAD prognosis using differentially expressed genes (DEGs) in the three molecular subtypes, and constructed a RiskScore that could predict immunotherapy effect and prognostic outcome of PAAD. The current results offered novel understanding for the development of feasible immunotherapy for PAAD.

## 2 Materials and methods

### 2.1 Dataset download and preprocessing

Gene chip expression profiles and corresponding clinical information for PACA-AU and PACA-CA sequencing projects were downloaded from the International Cancer Genome Consortium (ICGC, https://dcc.icgc.org/), respectively. In addition, the RNA-Seq data (TPM (transcripts per million)) and corresponding clinical information of the TCGA-PAAD cohort were acquired from The Cancer Genome Atlas (TCGA, https://portal.gdc.cancer.gov/) using the TCGA GDC application programming interface (API). The PACA-AU cohort served as the training set, and the PACA-CA and TCAG-PAAD cohorts served as the validation set. PACA-AU cohort were processed as follows: (Bengtsson et al., 2020): Removing samples that did not have clinical follow-up; (Skelton et al., 2017); Ensembl IDs were converted to a Gene symbol; (Siegel et al., 2019); Median expressions of multiple Gene Symbols was adopted. After screening, 267 PAAD samples were retained for subsequent analysis. RNA-Seq data in PACA-CA and TCGA-PAAD were processed as follows: (Bengtsson et al., 2020): PAAD samples without clinical information was removed; (Skelton et al., 2017); Ensembl IDs were converted to a Gene symbol; (Siegel et al., 2019); Expressions with multiple Gene Symbols adopted the middle value. After screening, 215 and 176 PAAD samples remained in the PACA-CA and TCGA-PAAD cohorts, respectively.

### 2.2 Evaluation of TME gene expression in PAAD

Based on previous studies, 29 TME gene sets representing major tumor functions and immune, stromal, and other cellular functions were downloaded from the Bagaev et al. study (Bagaev et al., 2021). Based on the expression data of 29 TME gene sets, Single-sample GSEA (ssGSEA) analysis was conducted on tumor samples in the PCA-AU cohort using the R package GSVA (Hanzelmann et al., 2013) to obtain the TME gene signature score. Subsequently, tumor samples and TME genes in the PACA-AU cohort were calculated for the signature correlation between scores. Finally, combined with the survival information of tumor samples from the PACA-AU cohort, we conducted univariate COX regression analysis to develop a TME gene signature affecting the prognosis of PAAD.

### 2.3 Identification of TME-associated molecular subtypes in PAAD

Based on the above 29 TME gene signature score, consistent clustering was conducted using R package ConsensusClusterPlus (Wilkerson and Hayes, 2010) in the PACA-AU cohort. In this process, 500 bootstraps were performed, with the “km” algorithm and “1-Pearson correlation” as the metric distance and each bootstrap processing 80% of the training set patients. Cluster number was from 2 to 10. The optimal classification was defined through consistency matrix calculation, and consistency cumulative distribution function was applied for obtaining PAAD molecular subtypes based on sample TME.

### 2.4 Association between PAAD molecular patterns with the clinical characteristics and prognosis

Clinical significance of the PAAD molecular subtypes was identified by consensus clustering, and we analyzed associations between molecular subtypes, clinical features (TNM Stage, Stage, Grade, Age, and Gender) and survival outcomes. The survminer (https://cran.r-project.org/web/packages/survminer/index. html) in R package survival (https://cran.r-project.org/web/packages/survival/index.html) was used for Kaplan-Meier survival analysis to assess differences in OS among different molecular subtypes.

### 2.5 Pathway characteristics between molecular subtypes

Gene Set Enrichment Analysis (GSEA) was conducted using the KEGG gene set (c2. cp.kegg.v7.4), with FDR <0.05 as a threshold to determine significantly enriched pathways (Subramanian et al., 2005).

### 2.6 Clinical significance of molecular subtypes and drug sensitivity analysis

T-cell–inflamed GEP is a predictor of response to pembrolizumab in cancer patients (Ayers et al., 2017). We calculated T-cell-Inflamed GEP score for each patient in different subtypes to predict pembrolizumab response in PAAD patients. Considering that IFN-γ is a critical cytokine in immune regulation and anti-cancer immunity, and that Th1 activates anti-tumor response of the body by biasing the secretion of IFN-γ, we also analyzed the Th1/IFNγ gene signature. Th1/IFNγ gene signature was from the study of Danilova et al. (Danilova et al., 2019), and the Th1/IFNγ score in each subtype sample was calculated using ssGSEA method. Next, we assessed differences in cytolytic activity and expression of CTLA4, LAG3, BTLA, HAVCR2, and TIGIT among different molecular subtypes. Finally, the half-inhibitory concentration (IC50) values of traditional chemotherapeutic agents applying the R package pRRophetic (Geeleher et al., 2014).

### 2.7 Programmed cell death (PCD) analysis

Twelve PCD patterns were acquired from previous (Zou et al., 2022). Altogether, 580 apoptosis genes, 52 pyroptosis genes, 87 ferroptosis genes, 367 autophagy genes, 14 cuproptosis genes, 9 parthanatos genes, 15 entotic cell death genes, 101 necroptosis genes, 8 netotic cell death genes, 7 alkaliptosis genes, 220 lysosome-dependent cell death genes, and 5 oxeiptosis genes were collected. Based on the expression data of above gene sets, ssGSEA analysis was conducted on PAAD tumor samples using the R package GSVA.

### 2.8 Identification and functional enrichment analysis of DEGs

We calculated DEGs between C1 vs. Other, C2 vs. Other, and C3 vs. Other using the R package limma (|log2FC| > 1 and FDR <0.05) (Ritchie et al., 2015). Based on these DEGs, the R package clusterProfiler was used for GO and KEGG enrichment analysis. (Yu et al., 2012).

### 2.9 Construction of PAAD prognostic risk assessment model

Univariate COX regression analysis on DEGs was performed in The R package for filtering genes showing greater impact on prognosis (*p* < 0.01). The R package glmnet (Simon et al., 2011) conducted to perform LASSO COX regression analysis, genes with strong correlation were removed after the selection of the best penalty parameter lambda using 10-fold cross-validation. Finally, using stepwise multivariate regression analysis, based on the Akaike Information Criterion (Portet, 2020), the DEGs with the smallest AIC value were selected as the key genes that affected the prognosis of PAAD, and a clinical prognosis prediction model was constructed. The formula is as follows:

$$RiskScore=\sum \beta_{i}*\mathrm{Exp}\;i$$

where i referred to the gene expression level, β was the corresponding gene Cox regression coefficient. Model performance was validated in the training set PACA-AU cohort and validation set cohorts PACA-CA, TCGA-PAAD cohorts.

### 2.10 Relationship between RiskScore grouping and TME and analysis of pathway characteristics

We performed GSEA enrichment analysis using the R package GSVA, and pathways with FDR <0.05 were considered as significantly enriched pathways. Next, the immune and stromal scores of PAAD patients were calculated with the ESTIMATE algorithm (Yoshihara et al., 2013) and we then determined the abundance of immune cell infiltration in 22 of each PAAD patient using the CIBERSORT algorithm (Chen et al., 2018). Finally, the relationship between RiskScore and cancer-related pathway activity score was studied.

### 2.11 Risk score group immunotherapy/chemotherapy difference analysis

Finally, differences in the effects of immunotherapy and chemotherapy between different Risk score groups were studied. We downloaded the TIDE scores of PAAD patient samples in the PACA-AU cohort from the TIDE (http://tide.dfci.harvard.edu/) database. A higher the TIDE prediction was positively correlated with a greater likelihood of immune escape, which indicate that the patients would be less likely to acquire immunotherapy benefit. Meanwhile, we downloaded the expression dataset IMvigor210 (http://research-pub.gene.com/IMvigor210CoreBiologies/) of 348 urothelial carcinoma patients treated with immunotherapy to verify the predictive significance of RiskScore.

### 2.12 Statistical analysis

R software (4.0.2, https://www.r‐project.org/) was used for statistical analysis. Rms package was used to build nomogram. The student’s t test was used to compare the two groups. Kruskal–Wallis test was used to compare the divergence between multiple groups. P< 0.05 considered as Statistical significance.

## 3 Result

### 3.1 The association between 29 TME gene signatures and clinical characteristics in PAAD patients

**FIGURE 1:**
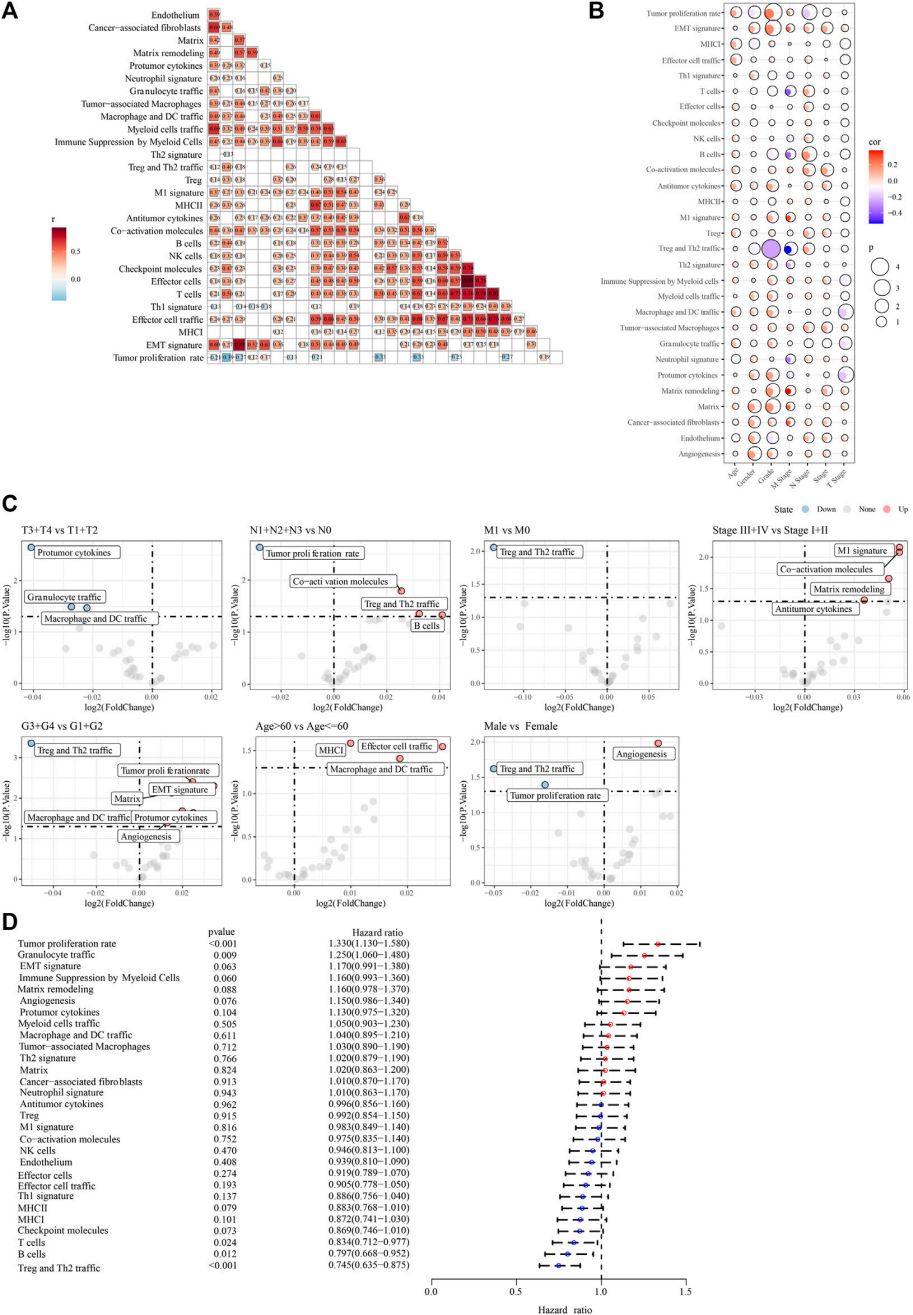
The association between 29 TME gene signatures and clinical characteristics in PAAD patients **(A)** correlation between TME of tumor samples in the PACA-AU cohort **(B)** correlation between TME and clinicopathological characteristics in the PACA-AU cohort **(C)** difference analysis in TME between different clinicopathological characteristics **(D)** forest map of univariate cox analysis results of TME signature.

The work flow was showed in Supplementary Figure S1. Based on 29 TME gene signatures, the TME gene signature score of the PACA-AU cohort samples were calculated using the ssGSEA enrichment analysis method. We found that most of the 29 TME gene signature scores were positively correlated with each other (Figure 1A). Next, the correlation of clinicopathological characteristics (gender, stage, age, TNM stage, Grade) and 29 TME gene signature scores of tumor patients were analyzed. Age was found to be positively correlated with most of the TME gene signature scores (Figure 1B). In addition, we also found that TNM Stage and Grade were positively correlated with EMT and with CAF-related TME signature (Figure 1B). Meanwhile, we also compared the differences in TME gene signature scores among the different pathological feature groups, and found that Co-activation molecules, Treg and Th2 traffic, and B cell scores were elevated in the N1+N2+N3 stage patients. M1 signature, Co-activation molecules, Matrix remodeling, and Antitumor cytokines were elevated in Stage III + IV patients. Matrix, Protumor cytokines, EMT signature, Tumor proliferation rate, and Angiogenesis were elevated in G3+G4 grade patients (Figure 1C). Finally, univariate COX regression analysis showed that Tumor proliferation rate (*p* < 0.001, HR = 1.330, 95%CI = 1.130–1.580), Granulocyte traffic (*p* = 0.009, HR = 1.250, 95%CI = 1.060–1.480), T cells (*p* = 0.024, HR = 0.834, 95%CI = 0.712–0.977), B cells (*p* = 0.012, HR = 0.797, 95%CI = 0.668–0.952) and Treg and Th2 traffic (*p* < 0.001, HR = 0.745, 95%CI = 0.635–0.875) and other TME gene signature scores were significantly associated with OS in PAAD patients (Figure 1D).

### 3.2 Identification of 3 molecular subtypes in PAAD

**FIGURE 2:**
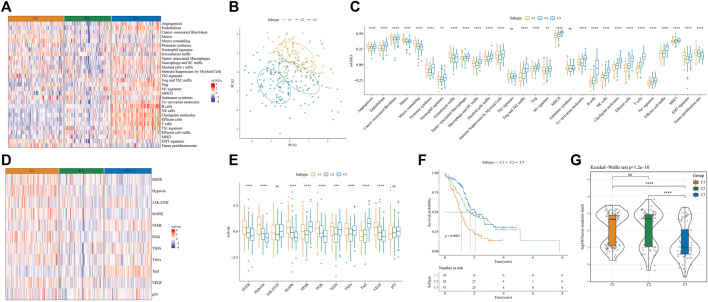
Analysis of TME differences in samples of 3 molecular subtypes divided by consistency clustering **(A)** heatmap of TME signature distribution in three molecular subtypes in the PACA-AU cohort **(B)** distribution of samples for PCA analysis of molecular subtypes **(C)** boxplots of TME signature score differences among three molecular subtypes in the PACA-AU cohort **(D)** Relative signaling pathway activity scores in tumor cells measured from RNA-seq by PROGENy **(E)** boxplots showing differences of relative signaling pathway activity scores in tumor cells measured from RNA-seq by PROGENy **(F)** Kaplan-Meier survival curves of three TME molecular subtypes **(G)** the difference of tumor mutation load among three molecular subtypes. *p < 0.05; **p < 0.01; ***p < 0.001; ****p < 0.0001; ns: no significance.

To classify TME subtypes in PAAD, we assessed the TME status of 267 PAAD patients in the PACA-AU cohort using 29 TME gene signature scores. Here, consistent clustering based on the TME gene signature score was used to classify patients with PAAD. Cumulative distribution function (CDF) determined the optimal cluster number was k = 3. Therefore, we obtained three molecular subtypes in PAAD, Cluster 1 (C1), Cluster 2 (C2) and Cluster 3 (C3) (Supplementary Figures S2A–C). The heatmap showed that the C1 subtype had significantly higher CAF, tumor-promoting gene signatures, and EMT and Tumor proliferation rate scores than the other two molecular subtypes, and the C3 subtype was significantly enriched for some immune-related signatures (Figure 2A). PCA analysis indicated that PAAD patients constituted molecular subtypes according to TME characteristics (Figure 2B). Furthermore, we found that except for Th2 signature and antitumor cytokines, the remaining 27 TME gene signature scores were significantly different among the three molecular subtypes (Figure 2C). In addition, we quantified the intensity of oncogenic signaling pathways using the expression of pathway target gene signatures. The results showed that signaling pathways such as EGFR, Hypoxia, MAPK, PI3K, VEGF were activated in the C1 subtype, and that NFκB, Trail signaling pathways were activated in the C3 subtype. (Figures 2D,E). Patients showed a better prognosis if with subtype C3, while that of those with subtype C1 was poorer, as shown by the results of survival analysis (Figure 2F). Moreover, the tumor mutation load was higher in C1 and C2 than that in C3 (Figure 2G) In general, based on the TME gene signature scores, the three molecular subtypes showed different immune activities, and the different TME status may influence the prognosis of PAAD patients.

### 3.3 Clinicopathological characteristics of the three PAAD molecular subtypes

**FIGURE 3:**
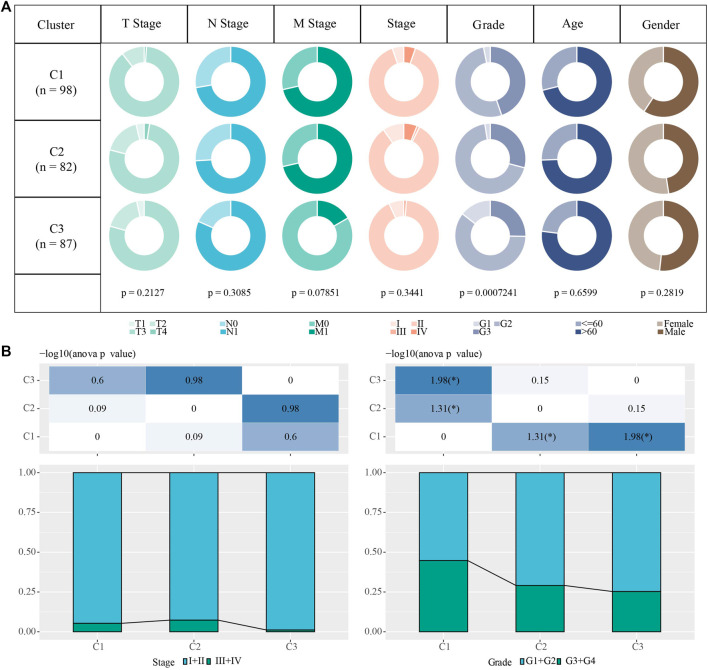
Clinicopathological characteristics of molecular subtype samples **(A)** clinicopathological characteristics of molecular subtypes in the PACA-AU cohort (Chi-square test) **(B)** clinicopathological characteristics of molecular subtypes in the PACA-AU cohort; The lower part is the proportion, and the upper part is the statistical significance of distribution difference between pairs (chi-square test).

In the PACA-AU cohort, we statistically analyzed the clinicopathological characteristics of Grade, Age, M stage, Gender, stage, T stage, N stage in patients of three subtypes to investigate the differences in clinicopathological characteristics of patients of different subtypes. The results showed that patients of C1 subtype had a higher clinical grading (Figures 3A, B).

### 3.4 Differences in biological pathways among molecular subtypes

According to the results of GSEA enrichment analysis, the C1 subtype in the PACA-AU cohort is mainly enriched in KEGG DNA replication, KEGG p53 signaling pathway, KEGG nucleotide excision repair, KEGG cell cycle-related pathways and EMT-related pathways such as KEGG focal adhesion and KEGG ECM receptor interaction (Supplementary Figure S3A). C2 Subtype are mainly enriched in KEGG retinol metabolism, KEGG metabolism of xenobiotics by cytochrome p450, KEGG glutathione metabolism, and other metabolism-related pathways (Supplementary Figure S3B). C3 Subtype are mainly enriched in KEGG cytokine KEGG complement and coagulation cascades, cytokine receptor interaction, KEGG primary immunodeficiency, KEGG antigen processing and presentation, KEGG chemokine signaling pathway in immune-related pathways (Supplementary Figure S3C). Meanwhile, the results of ssGSEA enrichment analysis showed that C1 Subtype were significantly enriched in hallmark PI3K/Akt/mTOR signaling, hallmark glycolysis, hallmark E2F targets, hallmark DNA repair, hallmark MYC targets v1, hallmark unfolded protein response, hallmark mitotic spindle hallmark G2M checkpoint, hallmark MYC targets v2, and other cell cycle related pathways, the C3 isoform is mainly enriched in hallmark allograft rejection, hallmark interferon alpha response, hallmark interferon gamma response, hallmark IL2/STAT5 signaling, hallmark IL6/JAK/STAT3 signaling, hallmark inflammatory response, and other immune-related pathways (Supplementary Figure S3D).

### 3.5 Immunotherapy/chemotherapy drug sensitivity/PCD analysis among molecular subtypes

**FIGURE 4:**
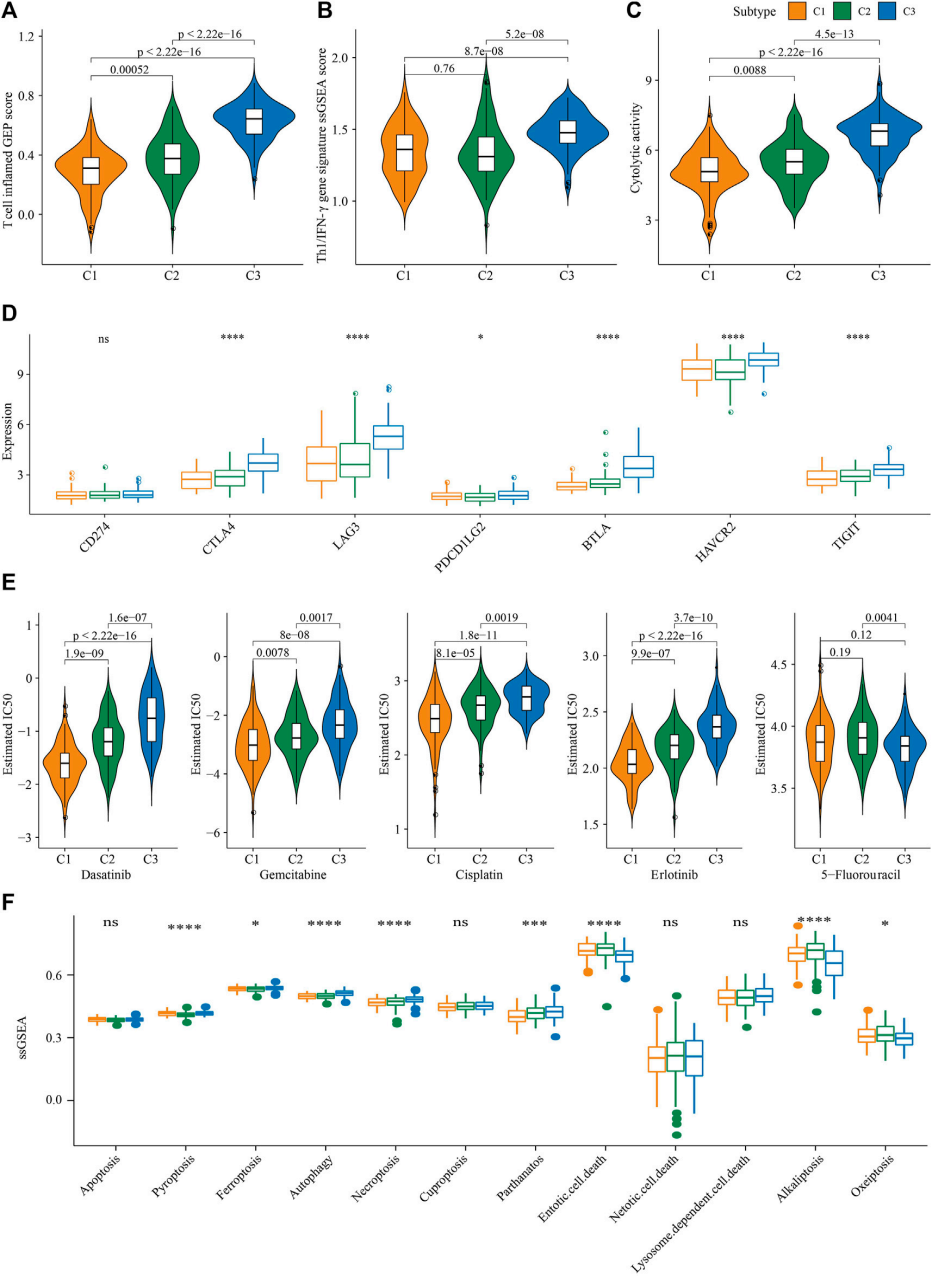
Immunotherapy/chemotherapy drug sensitivity analysis among molecular subtypes **(A)** T cell inflamed GEP score between different molecular subtypes **(B)** Th1/IFNγ gene signature ssGSEA score between different molecular subtypes **(C)** cytolytic activity between different molecular subtypes **(D)** Differential expression of immune checkpoint genes among different molecular subtypes **(E)** The boxplots of the estimated IC50 for dasatinib, gemcitabine, cisplatin, erlotinib and 5-Fluorouracil in PACA-AU **(F)** the ssGSEA score differences of 12 programmed cell death patterns among 3 molecular subtypes. *p < 0.05; ***p < 0.001; ****p < 0.0001; ns: no significance.

Though evaluating the T-cell–inflamed GEP score of patients with the 3 molecular subtypes in the PACA-AU cohort, we found that patients with the C3 subtype had a higher T-cell–inflamed GEP score (Figure 4A). The Th1/IFNγ gene signature ssGSEA score of the sample was calculated by the ssGSEA method, and the results show that the C3 subtype has a higher Th1/IFNγ gene signature ssGSEA score (Figure 4B). These results also implied that C3 subtype may be more sensitive to immunotherapy. In addition, cytolytic activeness as well as CTLA4, LAG3, BTLA, HAVCR2 and TIGIT were significantly higher in C3 subtypes than in C1 and C2 subtypes. (Figures 4C, D). Next, to determine the sensitivity of different subtypes of patients to chemotherapy, we assessed IC50 values for chemotherapeutics in patients in the PACA-AU cohort. It was found that the C1 subtype was more sensitive to the four chemotherapeutic agents Dasatinib, Gemcitabine, Cisplatin, and Erlotinib, while the C3 subtype was more sensitive to 5-Fluorouracil (Figure 4E). Combined with the results of TME analysis, C3 patients have higher TME activity and may be more adapted to immunotherapy, while C1 subtype patients have lower TME activity and were not sensitive to immunotherapy response. The ssGSEA analysis of 12 PCD patterns indicated that 8 PCD patterns had obviously differences among 3 subtypes, and in general, C2 or C3 subtype had higher ssGSEA scores (Figure 4F).

### 3.6 Internal validation and external exploration of TME molecular subtypes

**FIGURE 5:**
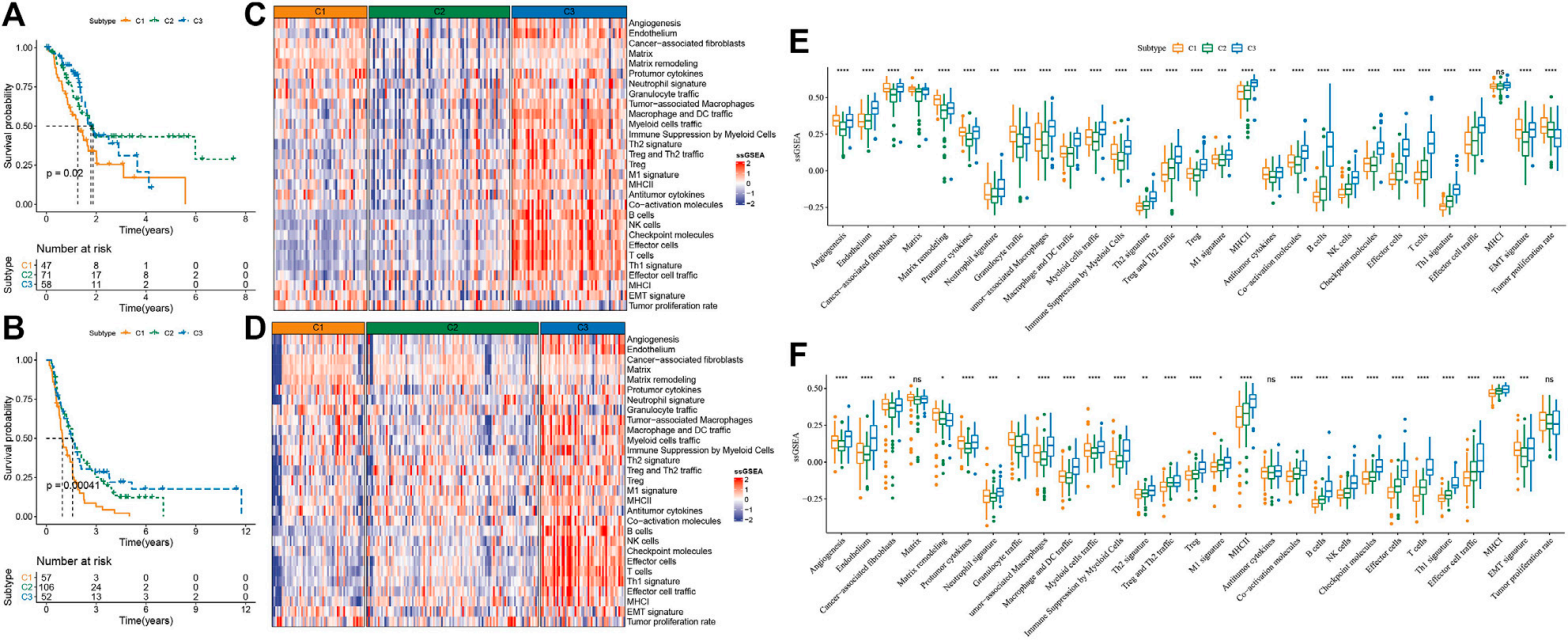
Internal validation and external exploration of tme clusters **(A)** Kaplan-Meier survival analysis between three different molecular subtypes in the TCGA-PAAD cohort **(B)** Kaplan-Meier survival analysis between three different molecular subtypes in the PACA-CA cohort **(C)** Distribution of TME signature among three molecular isoforms in the TCGA-PAAD cohort **(D)** Distribution of TME signature among three molecular subtypes in the PACA-CA cohort **(E)** Boxplot of differences in TME signature among the three molecular subtypes in the TCGA-PAAD cohort **(F)** Boxplot of differences in TME signature among the three molecular subtypes in the PACA-CA cohort. *p < 0.05; **p < 0.01; ***p < 0.001; ****p < 0.0001; ns: no significance.

Patients in the PACA-AU dataset were randomized into a training cohort (n = 215) and a test cohort (n = 52). We incorporated the TME score into the Artificial Neural Networks (ANN) model and validated it on the validation set to determine the TME clustering, where the hidden neural nodes of our ANN model were set to 5. The accuracy for the artificial neural network model in internal validation was 0.904, and that of the ANN model in the entire PACA-AU cohort was 0.981. In the external validation dataset, we obtained TME classification results using the SVM model and observed that the predicted TME clusters in the TCGA-PAAD and PACA-CA cohorts were consistent with the predicted survival differences in the PACA-AU dataset (Figures 5A, B). Furthermore, we also explored the landscape of infiltrating cells in the TME. Higher CAF scores were observed in C1 patients in different validation datasets. In addition, a higher immune infiltration can be observed in C3 patients. These findings in the validation dataset indicated that the pattern of enrichment of infiltrating cells was similar to the PACA-AU dataset (Figures 5C–F).

### 3.7 Differential expression analysis between molecular subtypes

**FIGURE 6:**
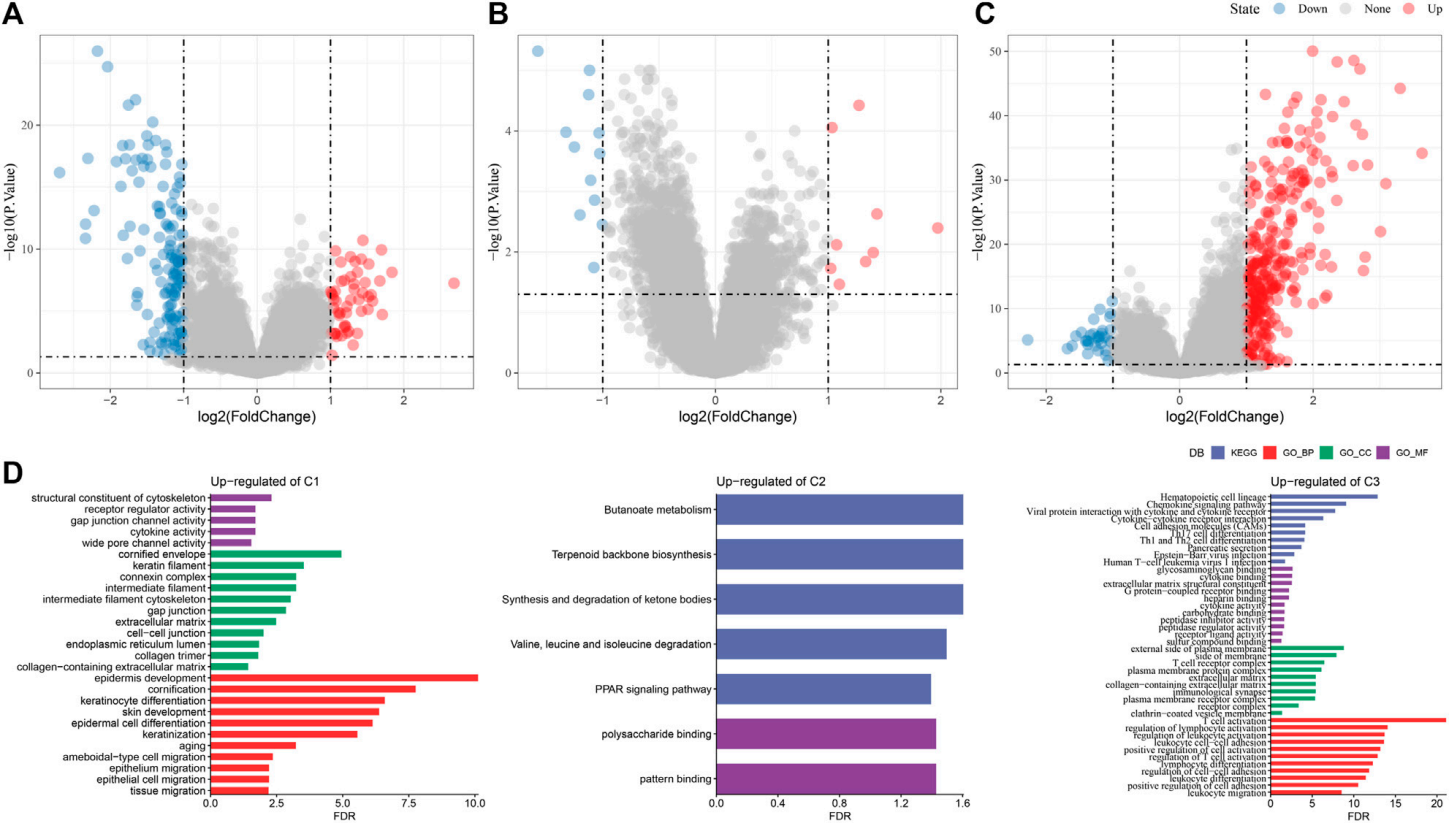
Identification of DEGs between molecular subtypes **(A)** volcano plot of DEGs in C1 vs. other in the PACA-AU cohort **(B)** volcano plot of DEGs in C2 vs. other in the PACA-AU cohort **(C)** volcano plot of DEGs in C3 vs. other in the PACA-AU cohort **(D)** functional enrichment analysis of upregulated DEGs in C1, C2 and C3 groups in the PACA-AU cohort.

To identify DEGs in the three PAAD molecular subtypes, we performed differential expression analysis for C1 vs. Other, C2 vs. Other and C3 vs. Other, respectively. Finally, 195 DEGs were identified in the C1 subtype, containing 53 upregulated genes and 142 downregulated genes (Figure 6A). 21 DEGs were identified in the C2 subtype, including 9 upregulated genes and 12 downregulated genes (Figure 6B). 339 DEGs were identified in the C3 subtype, including 307 upregulated genes and 32 downregulated genes (Figure 6C). GO and KEGG enrichment analysis on upregulated genes in C1, C2, and C3 subtypes showed that upregulated genes in C1 subtype were mainly enriched in the pathways related to cell matrix, and that the upregulated genes in the C2 subtype were significantly enriched to some metabolism-related pathways, moreover, upregulated genes in the C3 subtype were significantly enriched to some immune-related pathways (Figure 6D). The Pathway Interaction Database analysis showed that upregulated genes in C1 were enriched in HNF3A pathway, MYC repress pathway and ERA genomic pathway (Supplementary Figure S4A), upregulated gene in C3 were enriched in some inflammatory factor-related pathways (Supplementary Figure S4B). Wiki Pathways analysis indicated that upregulated genes in subtypes also enriched in some tumor related pathways, immune related pathways (Supplementary Figures S4C–E). Finally, 400 DEGs among the three molecular subtypes were screened.

### 3.8 Establishment and validation of clinical prognosis model

**FIGURE 7:**
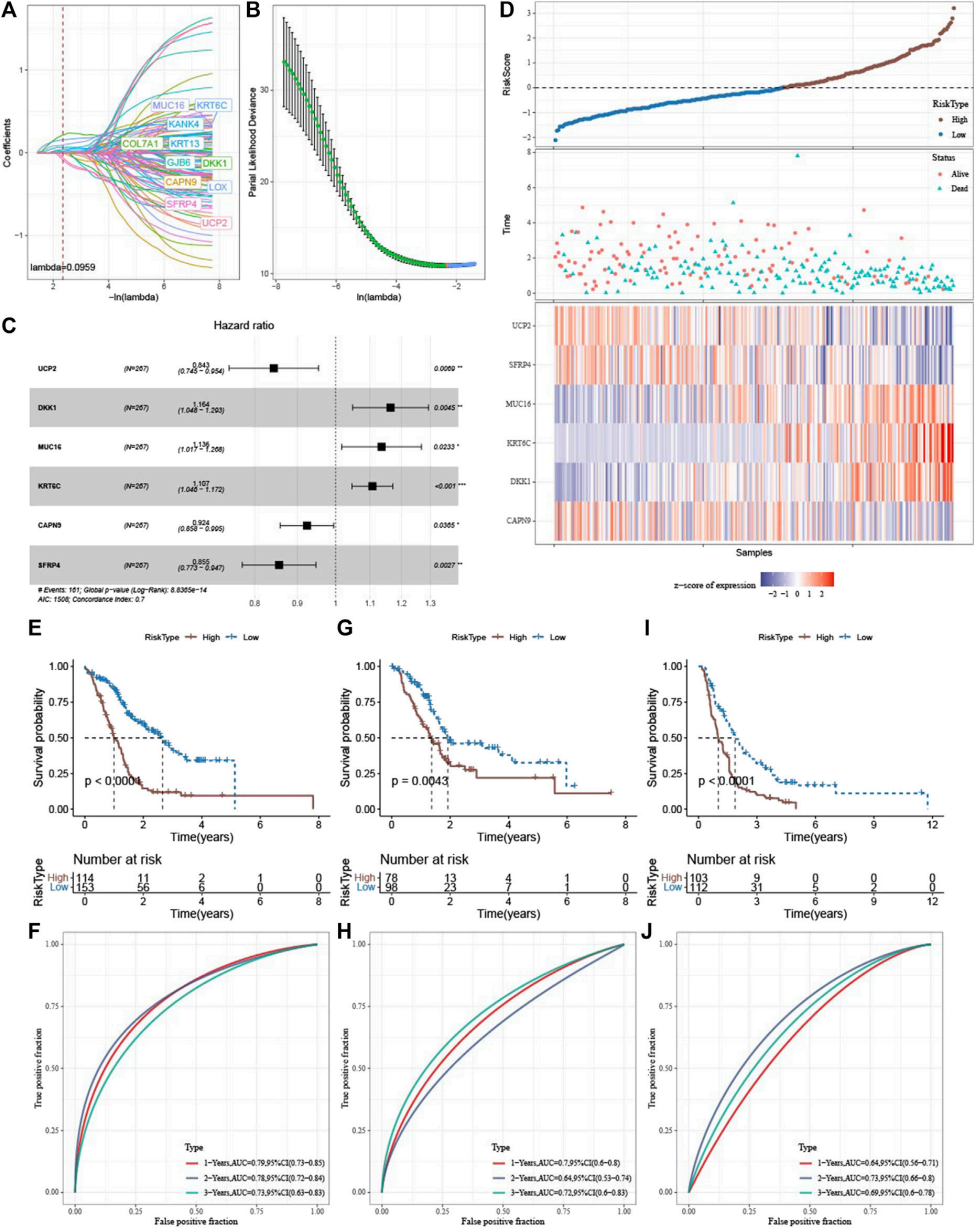
Establishment and validation of clinical prognostic model **(A)** LASSO coefficients of 11 genes in the PACA-AU cohort **(B)** determination of optimal lambda value using partial likelihood deviance of variables **(C)** Forest plot of multi-factor COX regression analysis **(D)** Sample distribution plot, survival status scatter plot, and 6-gene heatmap in the PACA-AU cohort **(E)** Kaplan-Meier survival analysis of high - and low-risk patients in the PACA-AU cohort **(F)** ROC curve of PACA-AU cohort **(G)** Kaplan-Meier survival analysis of high—and low-risk patients in the TCGA-PAAD cohort **(H)** ROC curve of TCGA-PAAD cohort **(I)** Kaplan-Meier survival analysis of high- and low-risk patients in the PACA-CA cohort **(J)** ROC curve of PACA-CA cohort.

To construct a clinical prediction model for PAAD, prognosis-related genes were screened from 400 DEGs. Model construction was performed using the PACA-AU cohort, and univariate COX regression analysis identified 110 genes from 400 DEGs with a high impact on OS in PAAD patients (*p* < 0.01), including 33 risk genes and 77 protective genes. LASSO COX regression analysis was conducted using these 110 genes, and the model was constructed by selecting the appropriate penalty parameter lambda with 10-fold cross-validation. We found that the model was optimal when lambda = 0959 with 11 genes including MUC16, KRT6C, KANK4, COL7A1, KRT13, GJB6, DKK1, CAPN9, LOX, SFRP4, UCP2(Figures 7A, B). Finally, multivariate COX regression analysis was further performed on these 11 genes, and the optimal model was selected based on the minimum AIC value with 6 genes (UCP2, DKK1, MUC16, KRT6C, CAPN9, SFRP4) as the PAAD prognostic gene. A risk assessment model was constructed, with HR of UCP2, CAPN9 and SFRP4 of less than 1 as protective factors and that of DKK1, MUC16, KRT6C greater than 1 as risk factors (Figure 7C). The RiskScore of each patient was calculated according to the following formula: RiskScore = 0.171 * UCP2 + 0.152 * DKK1 + 0.127 * MUC16 + 0.102 * KRT6C—0.079 * CAPN9—0.156 * SFRP4.

**FIGURE 8:**
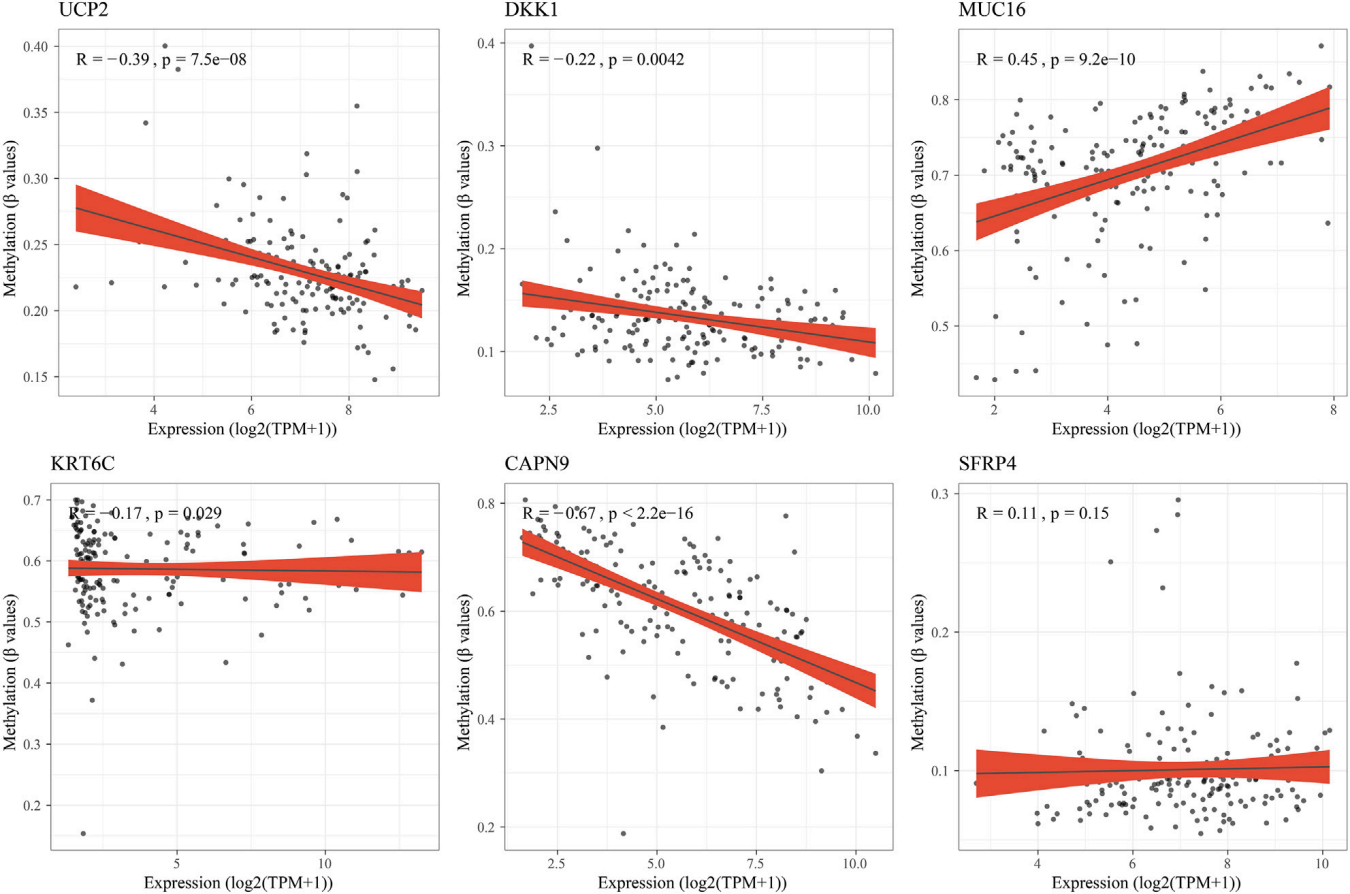
The correlation analysis between hub genes expressions and methylation level.

Subsequently, the RiskScore of tumor samples in the PACA-AU cohort was calculated and patients were divided into high-risk groups (n = 114) and low-risk groups (n = 153) with a threshold of 0. We found that PAAD patients had decreased survival time and increased mortality as their RiskScore increased. Meanwhile, the expression levels of protective factors such as UCP2, CAPN9, and SFRP4 in the low-risk group were significantly higher than those in the high-risk group, and the expression levels of risk factors such as DKK1, MUC16, and KRT6C in the high-risk group were significantly higher than those in the low-risk group (Figure 7D). Subsequently, we plotted the survival curves between high- and low-risk groups, and found that patients in the high-risk group had a poorer prognosis in contrast to those in the low-risk group with a better prognosis (Figure 7E). Prediction efficiency for the RiskScore was assessed by ROC analysis, showing an AUC value of 0.79, 0.78, and 0.73 at 1-, 2-, and 3-year (s), respectively (Figure 7F). To further validate the predictive accuracy of the clinical prognostic model, we performed validation in external independent validation sets PACA-CA and TCGA-PAAD. The results were consistent with the PACA-AU cohort, because poor prognosis in the high-risk group and better prognosis in the low-risk group were observed. The ROC analysis of the PACA-CA cohort showed that the AUC values were 0.7, 0.64, and 0.72 at 1-, 2-, and 3-year(s), respectively, and the ROC analysis of the TCGA-PAAD cohort showed that at 1-, 2- year and 3-year AUC value was 0.64, 0.73, and 0.69, respectively (Figures 7G–J). These results suggested that our PAAD clinical prognostic model based on DEGs among different molecular subtypes had a high predictive accuracy. Furthermore, we found 5 of 6 model genes expressions were closely associated with methylation level of those genes (Figure 8).

### 3.9 RiskScore performance on different clinicopathological characteristics and different molecular subtypes

**FIGURE 9:**
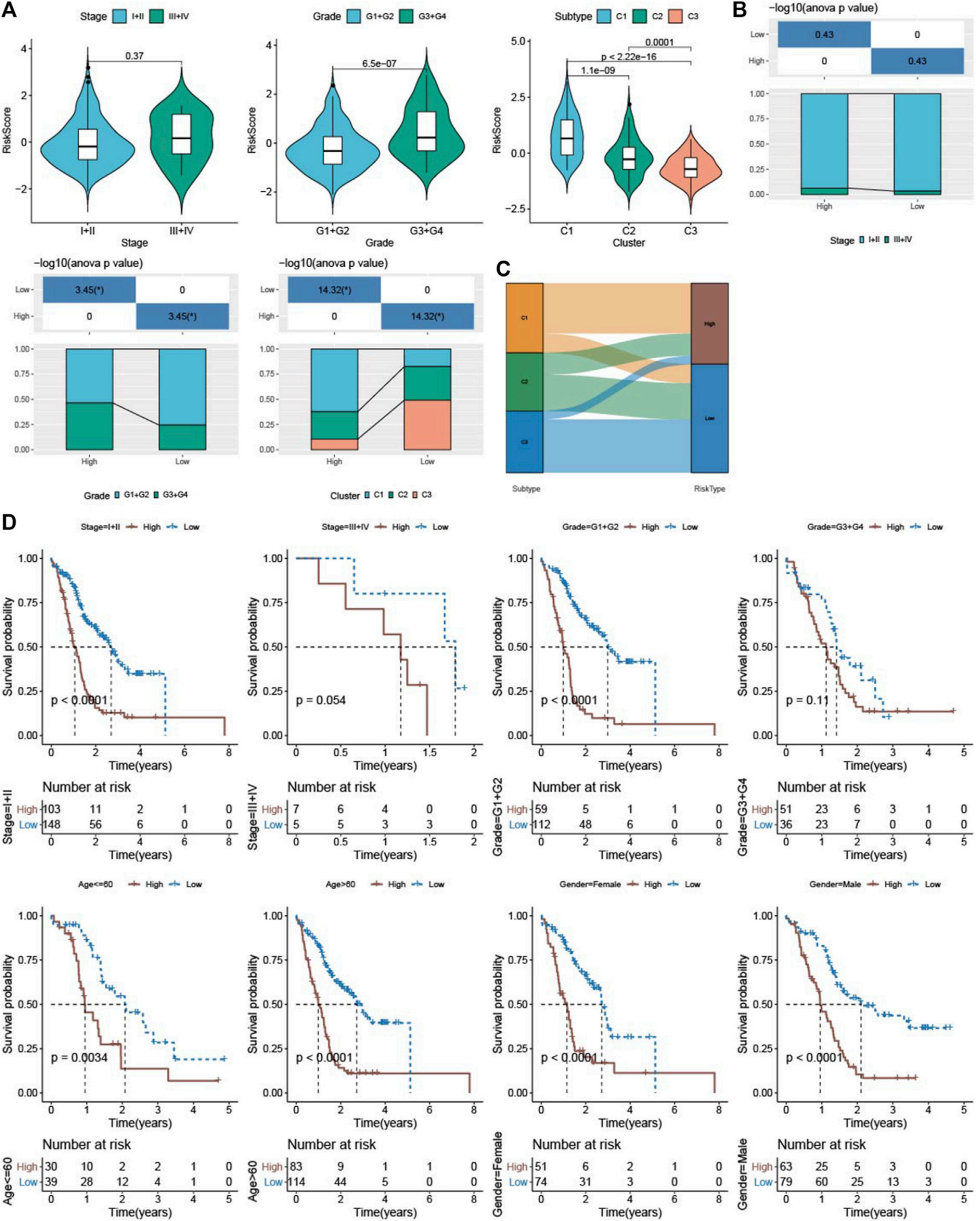
Association of riskscore with clinicopathological characteristics and molecular subtypes **(A)** Differences in riskscore between different clinicopathological groupings and between TME molecular subtypes in the PACA-AU cohort **(B)** clinicopathological information in the high and low risk group of the PACA-AU cohort; The lower part is the proportion, and the upper part is the statistical significance of the distribution difference between the two pairs **(C)** Sankey diagram of subtype distributions in groups with different RiskScore **(D)** Kaplan-Meier survival analysis between different clinicopathological groups in the PACA-AU cohort with high and low RiskScore.

To examine the relationship between RiskScore and clinicopathological characteristics of PAAD, we analyzed the differences in RiskScore between different TNM grades and Stage clinical grades in the PACA-AU dataset. RiskScore of patients increased with clinical grade, and interestingly, and patients with C1 subtype were significantly higher than C2 and C3 subtypes (Figure 9A). Notably, those in the high-risk group had a higher clinical grade and a higher proportion of patients with the C1 molecular subtype (Figure 9B). Meanwhile, the result was further verified by the Sankey diagram (Figure 9C). Finally, survival analysis in different clinicopathological characteristics subgroups revealed that patients with low RiskScore had higher survival rates in different clinical subgroups (Figure 9D). These results further demonstrated that the clinical prognostic model of PAAD constructed in this study was a reliable predictor.

### 3.10 Immune infiltration/pathway characteristics between two groups

**FIGURE 10:**
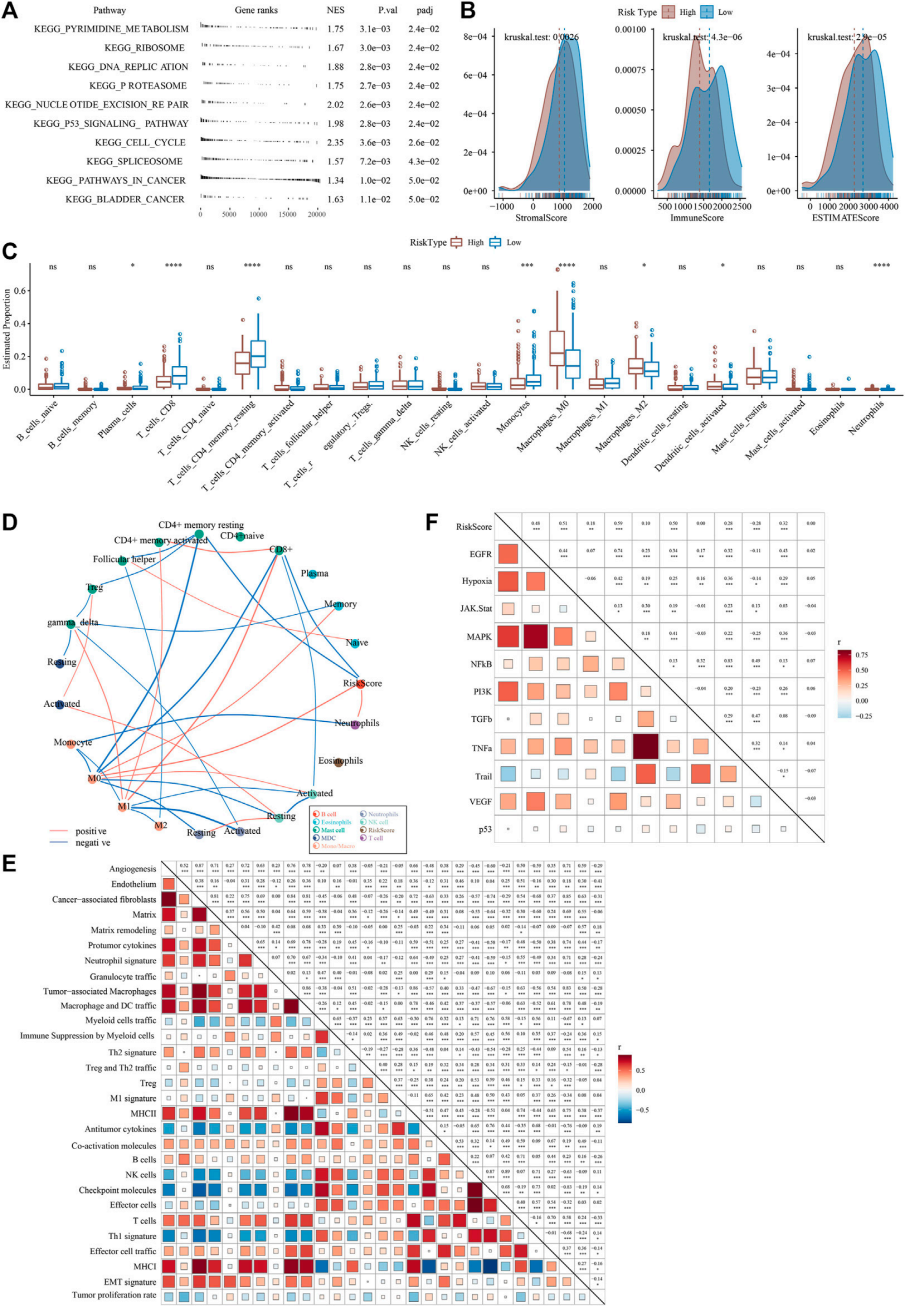
RiskScore performance on different clinicopathological characteristics and different molecular subtypes **(A)** A GSEA algorithm was performed with all KEGG gene sets in different RiskScore groups **(B)** ESTIMATE analysis between high- and low-risk groups in the PACA-AU cohort **(C)** abundance of 22 types of immune cell infiltration **(D)** correlation analysis of 22 immune cell components with RiskScore **(E)** correlation between riskscore and TME gene signatures ssGSEA score **(F)** Correlation between RiskScore and pathway activity scores. *p < 0.05; **p < 0.01; ***p < 0.001; ****p < 0.0001; ns: no significance.

To explore the differences in biological pathways in the high- and low-risk groups, GSEA enrichment analysis was performed. We found that the high-risk group was significantly enriched in cell cycle-related signaling pathways such as KEGG_P53_SIGNALING_PATHWAY and KEGG_CELL_CYCLE (FDR <0.05) (Figure 10A). ESTIMATE analysis showed that the low-risk group had higher StromalScore, ImmuneScore, and ESTIMATEScore (Figure 10B). In addition, we also used the CIBERSORT algorithm to calculate the abundance of 22 types of immune cells in patients in the two risk groups. It was observed that the infiltration levels of T_cells_CD8 and T_cells_CD4_memory_resting in the low-risk group were significantly higher than those in the high-risk group (Figure 10C). Analysis on RiskScore with the level of 22 types of immune cell infiltration showed that the RiskScore was positively correlated with Neutrophils abundance and negatively correlated with CD8^+^ T cell, CD4^+^ menory resting T cell abundance (Figure 10D). A significant positive correlation was found between RiskScore and gene signatures related to Angiogenesis and Fibroblasts and EMT (Figure 10E). Finally, RiskScore was found to be significantly positively correlated with EGFR, Hypoxia, MAPK, PI3K, and TNFa and VEGF pathways (Figure 10F).

### 3.11 Differences in immunotherapy/chemotherapy/PCD patterns between riskscore groups

**FIGURE 11:**
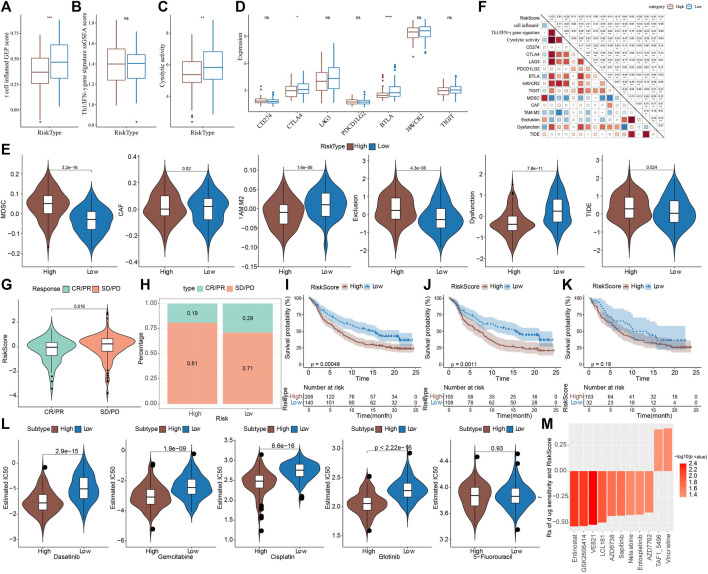
Differences in immunotherapy/chemotherapy between riskscore groups **(A)** differences in T cell inflamed GEP scores between high and low risk groups **(B)** differences in Th1/IFNγ gene signature ssGSEA score between high- and low-risk groups **(C)** differences in cytolytic activity between high- and low-risk groups **(D)** differences in immune checkpoint expression between high- and low-risk groups **(E)** Differences in TIDE between high- and low-risk groups **(F)** Correlation analysis of RiskScore with T cell inflamed GEP score, Th1/IFNγ gene signature ssGSEA score, Cytolytic activity, immune checkpoint gene expression and TIDE **(G)** RiskScore differences among samples in the IMvigor210 cohort **(H)** Distribution of immunotherapy response between high- and low-risk groups in the IMvigor210 cohort **(I)** Kaplan-Meier survival analysis between high and low risk groups in the IMvigor210 cohort **(J)** Kaplan-Meier survival analysis between high- and low-risk groups for early Stage I + II patients in the IMvigor210 cohort **(K)** Kaplan-Meier survival analysis between high- and low-risk groups for early Stage III + IV patients in the IMvigor210 cohort **(L)** The box plots of the estimated IC50 for docetaxel, vinorelbine, paclitaxel and cisplatin in PACA-AU **(M)** Relationship between RiskScore and drug sensitivity. *p < 0.05; **p < 0.01; ***p < 0.001; ****p < 0.0001; ns: no significance.

Next, the efficacy of RiskScore as a biomarker in predicting immunotherapy and chemotherapy response of PAAD patients was further validated. First, we evaluated the T-cell-inflamed GEP score and Th1/IFNγ gene signature ssGSEA score in the high-risk and low-risk groups, and the results showed that the T-cell-inflamed GEP score, not Th1/IFNγ gene signature score, was higher in the low-risk group (Figures 11A, B). Meanwhile, cytolytic activity, CTLA4 and BTLA were higher in the low-risk group (Figures 11C, D). We also found that MDSC, Exclusion and TIED scores were lower in the low-risk group, indicating that patients in the low-risk group may be more sensitive to immunotherapy (Figure 11E). Further, we analyzed the correlation between RiskScore and T-cell-inflamed GEP score, Th1/IFNγ gene signature score, immune checkpoint expression, and TIDE. It was clearly observed that RiskScore was significantly positively correlated with MDSC, Exclusion, and TIDE but negatively correlated with T cell inflamed GEP score, Cytolytic activity and the expression of immune checkpoint gene (Figure 11F).

Furthermore, to test the ability of our model in predicting patients’ benefit from immunotherapy, we applied the model to the immunotherapy cohort (IMvigor210 cohort). In the IMvigor210 cohort, anti-PD-L1 treatment of patients with progressive disease (PD) and stable disease (SD) had a significantly higher RiskScore than the complete remission (CR) and partial remission (PR) groups (Figure 11G). We also found a higher proportion of CR/PR patients in the low-risk group (Figure 11H). In addition, patients in the high-risk group had a significantly shorter survival time than those in the low-risk group (Figure 11I). In Stage I + II stage patients, the survival time of patients in the high-risk group was also significantly shorter than that in the low-risk group, while the difference was not significant in Stage III + IV (Figures 11J, K). These findings suggested that RiskScore can be used to predict immunotherapy response in PAAD patients, and importantly, RiskScore is a reliable predictor in the prognosis prediction of early PAAD patients. Finally, in the PACA-AU cohort, we observed that patients in the high-risk group were more sensitive to Dasatinib, Gemcitabine, Cisplatin, Erlotinib (Figure 11L). Then, Pearson correlation analysis was conducted between RiskScore and sensitivity of chemotherapy drugs, and it was observed that RiskScore was significantly correlated with sensitivity of 11 drugs, and significantly negatively correlated with Entinostat, GSK2606414, VE821, LCL161, AZD6738, Sapitinib, Nelarabine, Entospletinib, AZD7762. RiskScore was positively correlated with TAF1_5496 and Vincristine. (Figure 11M). These results demonstrated that RiskScore was potentially a reliable biomarker for predicting PAAD patients’ response to immunotherapy and chemotherapy.

**FIGURE 12:**
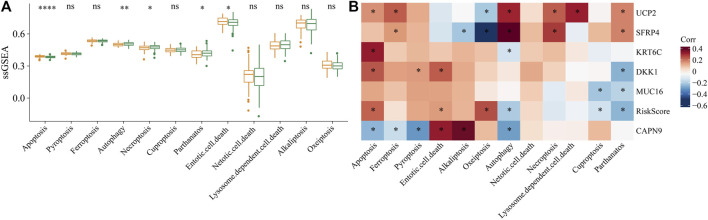
The association between programmed cell death patterns and RiskScore. **(A)** The ssGSEA score differences of 12 programmed cell death patterns between high- and low-group. **(B)** The correlation analysis between 12 programmed cell death patterns and RiskScore. *p < 0.05; **p < 0.01; ****p < 0.0001; ns: no significance.

In addition, 5 of 12 PCD patterns had increased ssGSEA score in low group in comparison to high group (Figure 12A). Further, we analyzed the correlation between RiskScore, 6 model genes and 12 PCD patterns, and there were different degrees of correlation with each other (Figure 12B).

### 3.12 Construction of nomogram

**FIGURE 13:**
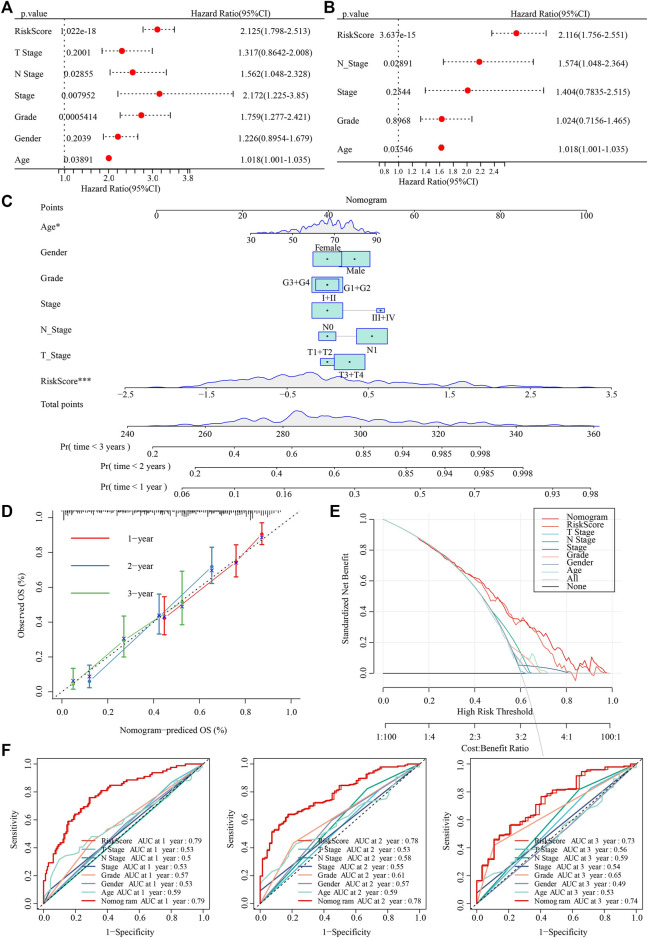
Establishment of nomogram. **(A,B)** Univariate and multivariate cox regression analysis of RiskScore and clinical features. **(C)** A nomogram was developed using riskScore and clinical features. **(D)** calibration plots of standard curve and actual forecast curve. **(E)** decision curve analysis. **(F)** AUC analysis of riskscore, nomogram, clinical features.

Univariate and multivariate cox regression analysis showed that RiskScore, N stage and Age were independent prognostic factors (Figures 13A, B). Nomogram combining RiskScore and clinical features was used to predict the probability of 1-, 2- and 3‐years OS in PAAD patients (Figure 13C). Moreover, calibration plots indicated that in comparison with an ideal model, the nomogram had a similar performance (Figure 13D). The results of DCA also indicated that our nomogram had high potential for clinical utility (Figure 13E). Moreover, nomogram had higher AUC in 1-, 2- and 3-year (Figure 13F).

## 4 Discussion

Immunotherapy as a new and promising cancer treatment modality kills tumor cells by enhancing antitumor response of tumor patients (Yang, 2015). Excitingly, immunotherapy that enhances immune cell—mediated tumor killing by inhibiting immune checkpoints has been shown to be a promising treatment for melanoma and colorectal cancer. However, patients with some solid tumors, including PAAD, could hardly benefit from immunotherapy (McCormick et al., 2016). Tumor microenvironment has been well known to play a critical role in clinical outcomes and treatment response of cancer by affecting immune cell infiltration, tumor progression, and drug antitumor effects (Xiao and Yu, 2021). Although the importance of TME in cancer is well established, PAAD lacks comprehensive analysis of transcriptomic data based on the combination of tumor and TME. Therefore, mining TME signature and clinical treatment response for PAAD patients is crucial for elucidating its pathogenic mechanism and developing new therapeutic strategies.

In this study, PAAD samples were described as three different TME molecular subtypes (C1, C2, C3) using consistent clustering based on ssGSEA scores of 29-TME signature in the PACA-AU cohort. Among them, the signature of CAF and tumor-promoting association in C1 subtype showed a higher score. CAF is the most abundant cell in stromal cells in TME and it regulates the biological properties of cancer cells and other stromal cells through coordinating crosstalk within TME and releasing multiple regulatory factors (Chen et al., 2021a). The extracellular matrix reconstituted by CAFs could act as a physical barrier to support tumor cell infiltration and inhibit antitumor leukocyte infiltration, resulting in immune escape, cancer progression, and immunotherapy resistance (Kaur et al., 2019). Furthermore, by impairing drug delivery and immune signaling pathways, CAF may confer actual therapeutic resistance (Kalluri, 2016). Interestingly, a previous study showed that high CAF leads to poor prognosis in cancer patients (Pelon et al., 2020). Survival analysis in the current study showed that patients with C1 molecular subtype had higher CAF signature and clinicopathological stage, and that those with C1 molecular subtype had the worst prognosis. This was also in accordance with previous studies. Similarly, patients with high clinical grade was significantly more in the C1 molecular subtype, which was less sensitive to immunotherapy. We speculated that this may be due to immunotherapy resistance resulted from high CAF infiltration.

From the three molecular subtypes, we screened UCP2, DKK1, MUC16, KRT6C, CAPN9, SFRP4 and other genes related to PAAD prognosis and constructed a clinical prognosis model. UCP2 was found to function crucially in pancreatic ductal adenocarcinoma (PDAC), and that UCP2 silencing reduces glutaminolysis and nicotinamide adenine dinucleotide phosphate (NADPH) production in P DAC cell lines, ultimately affecting tumor growth (Raho et al., 2020). In breast cancer metastasis, DKK1 is a serum marker that promotes breast cancer bone metastasis by regulating the WNT signaling pathway (Zhuang et al., 2017). Moreover, MUC16 is associated with PDAC malignant progression, and that truncated O-glycans containing MUC16 activate FAK signaling through specific interactions with α4 and β1 integrin complexes on cancer cell membranes, contributing to PDAC malignancy progress (Rajesh et al., 2022). The study found that for high-grade serous ovarian cancer (HGSOC), CAPN9 may be a therapeutic target. By binding to the 3′-untranslated region (UTR) of CAPN9, tumor suppressor miR-585-3p inhibits the expression of CAPN9 to suppress the growth and migration of HGSOC (Lu et al., 2021). High-expressed SFRP4, which correlates with a poor prognosis of gastric cancer, can activate the Wnt pathway to promote tumor progression and predict a poor survival of patients suffering from gastric cancer (Li et al., 2021). However, KRT6C is less studied in cancer, and its specific mechanism is still unclear. In this study, our clinical prediction model composed of 6 signature genes showed excellent predictive performance. Combined with published studies, most of the PAAD prognostic signature genes we identified are associated with tumor malignant progression. We speculated that the six genes may be new therapeutic targets for PAAD, but its molecular mechanism in PAAD should be further explored in order to understand its role in tumor progression and treatment.

In this study, patients were divided by the RiskScore, and those with a low risk survived longer. This was also verified in the external validation sets TCGA-PAAD and PACA-CA. Notably, PAAD patients with poor prognosis C1 subtype or higher subtype were assigned to the high-risk group, which might be explained by different TME states in PAAD. The infiltration level of Macrophages_M0 and Macrophages_M2 was higher in the high-risk group, while that of T_cells_CD8 and Monocytes was higher in the low-risk group. CD8 + T cells are the main killer of tumor cells, and factors such as C AF accumulated in TME can lead to depletion of CD8 + T cells, which in turn cause immune escape of tumor cells (Raskov et al., 2021). Chen and colleagues found that polarization of monocyte-derived macrophages into M2 macrophages could be induced by both CAFs and cancer cells, thereby promoting HCC cells’ malignant features (Chen et al., 2021b). In contrast, M2 macrophages stimulate tumor growth by promoting tumor immunosuppression (Pan et al., 2020). This was supported by our current findings, and we hypothesized that higher levels of CAF may deplete CD8^+^ T cells and induce Macrophages_M0 in high-risk patients to M2 polarization, eventually leading to immune evasion and the development of high-grade tumors. Furthermore, in the IMvigor210 cohort of patients receiving anti - PD-L1 therapy, RiskScore could predict PAAD patients’ response to ICB therapy. These findings suggest that the RiskScore can assess the prognostic status and immunotherapy response of PAAD patients based on their TME status, and was therefore an efficient and accurate biomarker that could be expected to provide important insights for guiding clinical practice.

Collectively, we have identified molecular subtypes of TME signatures and constructed a predictive model of PAAD clinical prognosis based on transcriptomic and clinical data from public databases of PAAD. Patients with high- and low-RiskScore showed different immune infiltration and survival rates. Functionally, RiskScore has excellent ability to predict the response to immunotherapy and chemotherapeutic drug sensitivity in PAAD patients. The model has good prognostic accuracy and could promote individualized prognostic management and individualized therapeutic intervention. Combined with clinical characteristics and risk characteristics, the prediction performance of PAAD was further improved. And it is worthy of further *in vitro* and *in vivo* studies. However, there were still some deficiencies in this study. Firstly, we lack detailed treatment data of PAAD—related immunotherapy cohorts. The RiskScore was validated in the IMvigor210 cohort (immunotherapy cohort for urothelial carcinoma) for the immunotherapy of PAAD patients. Secondly, this study used only research data in public databases for bioinformatics analysis, and basic or clinical experiments were need to validate the actual clinical utility of RiskScore in evaluating PAAD. Therefore, follow-up multi-center large-scale clinical trials are needed to verify the actual clinical value of RiskScore in assessing the prognosis and immunotherapy response of PAAD.

## 5 Conclusion

We developed TME signatures and constructed a predictive model of PAAD clinical prognosis based on transcriptomic and clinical data from public databases of PAAD. Different PAAD molecular subtypes and RiskScore patients showed different prognosis, pathological characteristics, immune characteristics and immunotherapy response. RiskScore was an efficient and accurate biomarker that could be expected to provide important insights for clinical practice guidance.

## Data Availability

The original contributions presented in the study are included in the article/Supplementary Material, further inquiries can be directed to the corresponding author.

## Abbreviations

AUC, area under the curve; BP, biological process; CC, cellular component; CNV, copy number variation; CSC, cancer stem cell; DEG, differentially expressed gene; DCA, decision curve analysis; EMT, epithelial-mesenchymal transformation; GEO, gene expression omnibus; GSEA, Gene set enrichment analysis; GO, gene ontology; HCC, hepatocellular carcinoma; HBV, hepatitis B virus; HCV, hepatitis C virus; LASSO, least absolute shrinkage and selection operator; MF, molecular function; OS, overall survival; PTEN, Phosphatase and tensin homologue; ROC, Receiver operating characteristic; SsGSEA, single-sample gene set enrichment analysis; TCGA, the cancer genome Atlas; TME, tumor microenvironment; TIL, tumor-infiltrating lymphocyte; TIDE, tumor immune dysfunction and ejection.
