# Supplementary figures and images for: Characterization of tumor microenvironment and programmed death-related genes to identify molecular subtypes and drug resistance in pancreatic cancer
Source: Front Pharmacol. 2023 Mar 17;14:1146280. doi: 10.3389/fphar.2023.1146280 (PMC10063807; doi:10.3389/fphar.2023.1146280)

A

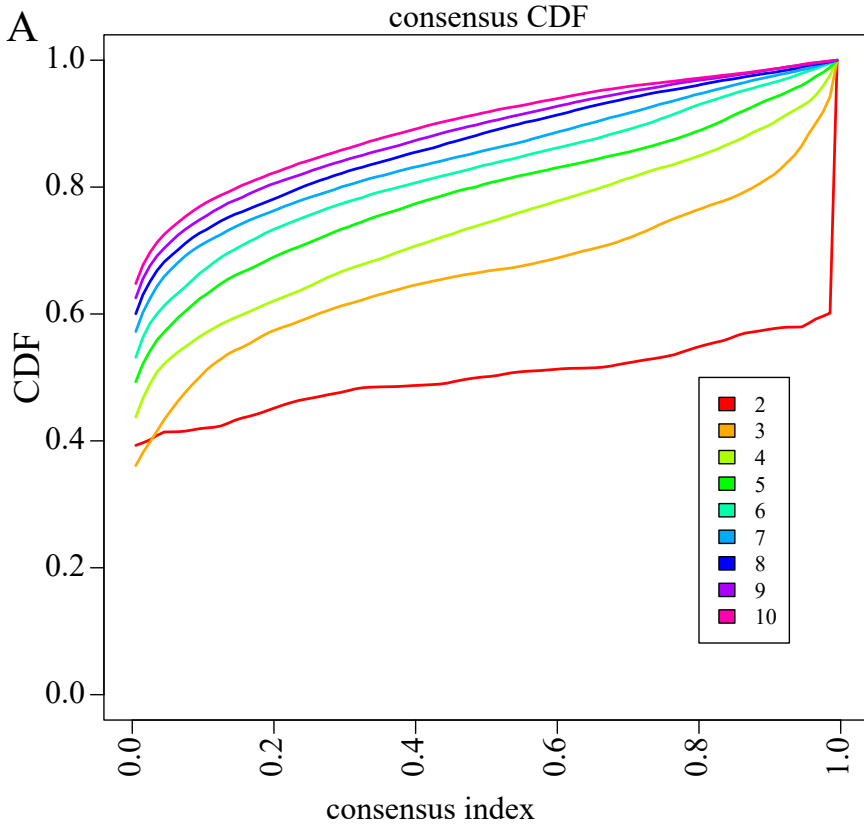

B

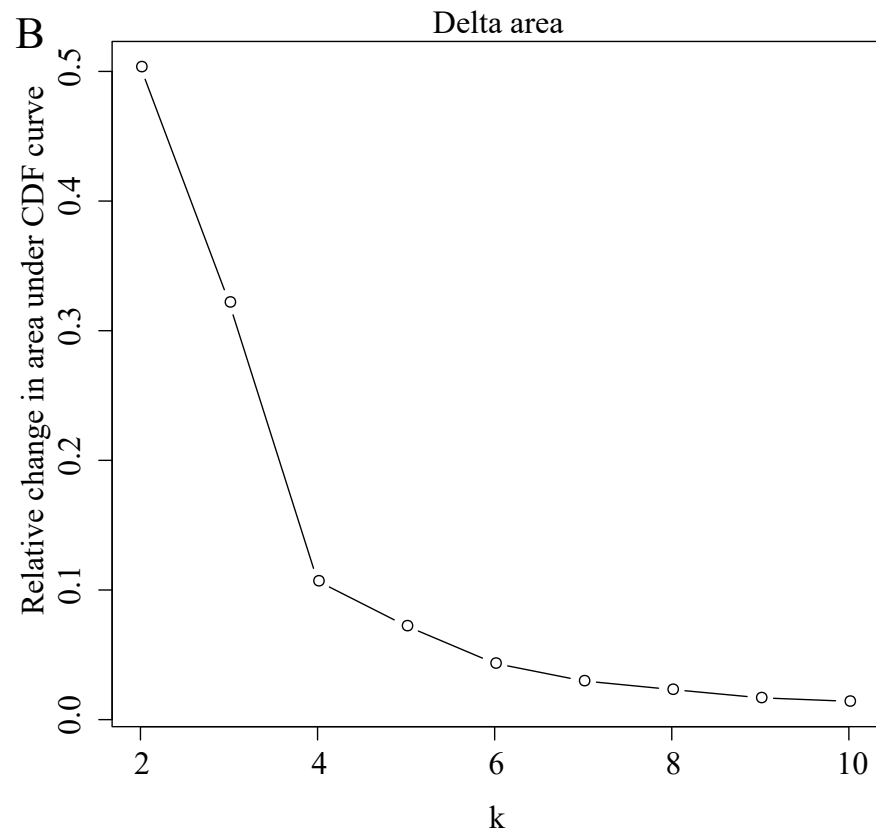

C

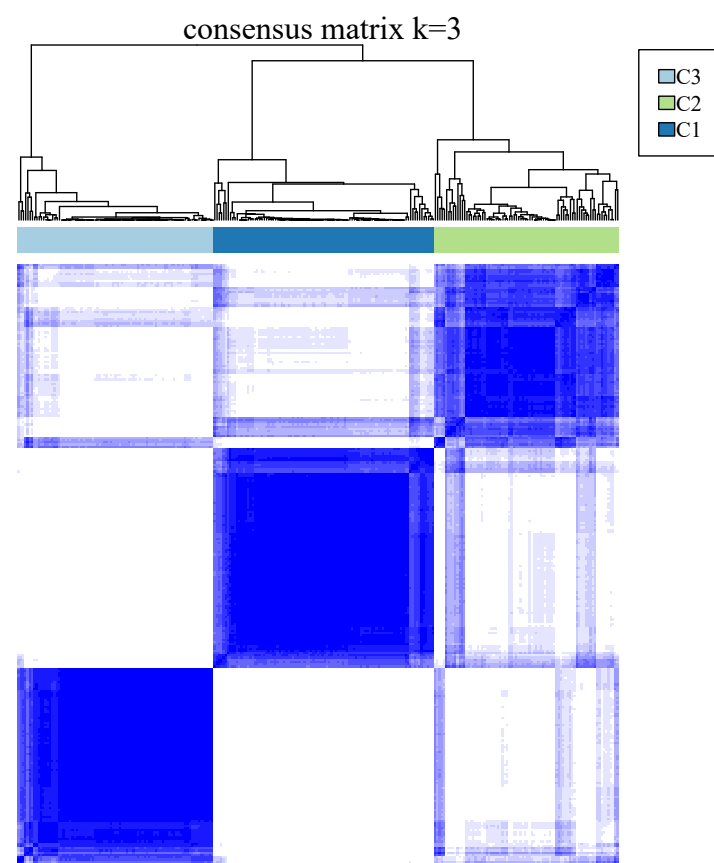

Supplement: Supplementary file 1 [file DataSheet2.PDF]

A

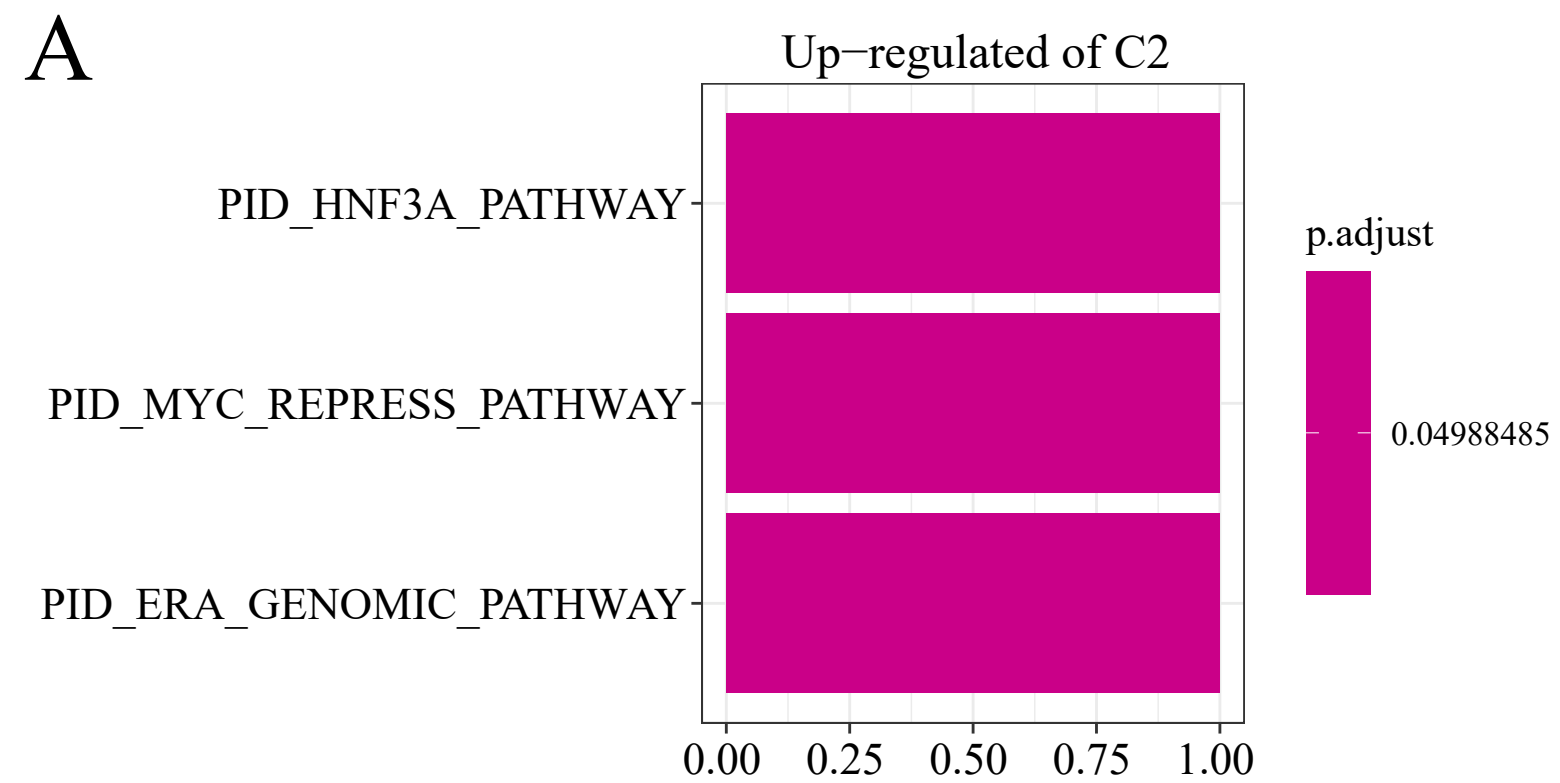

B

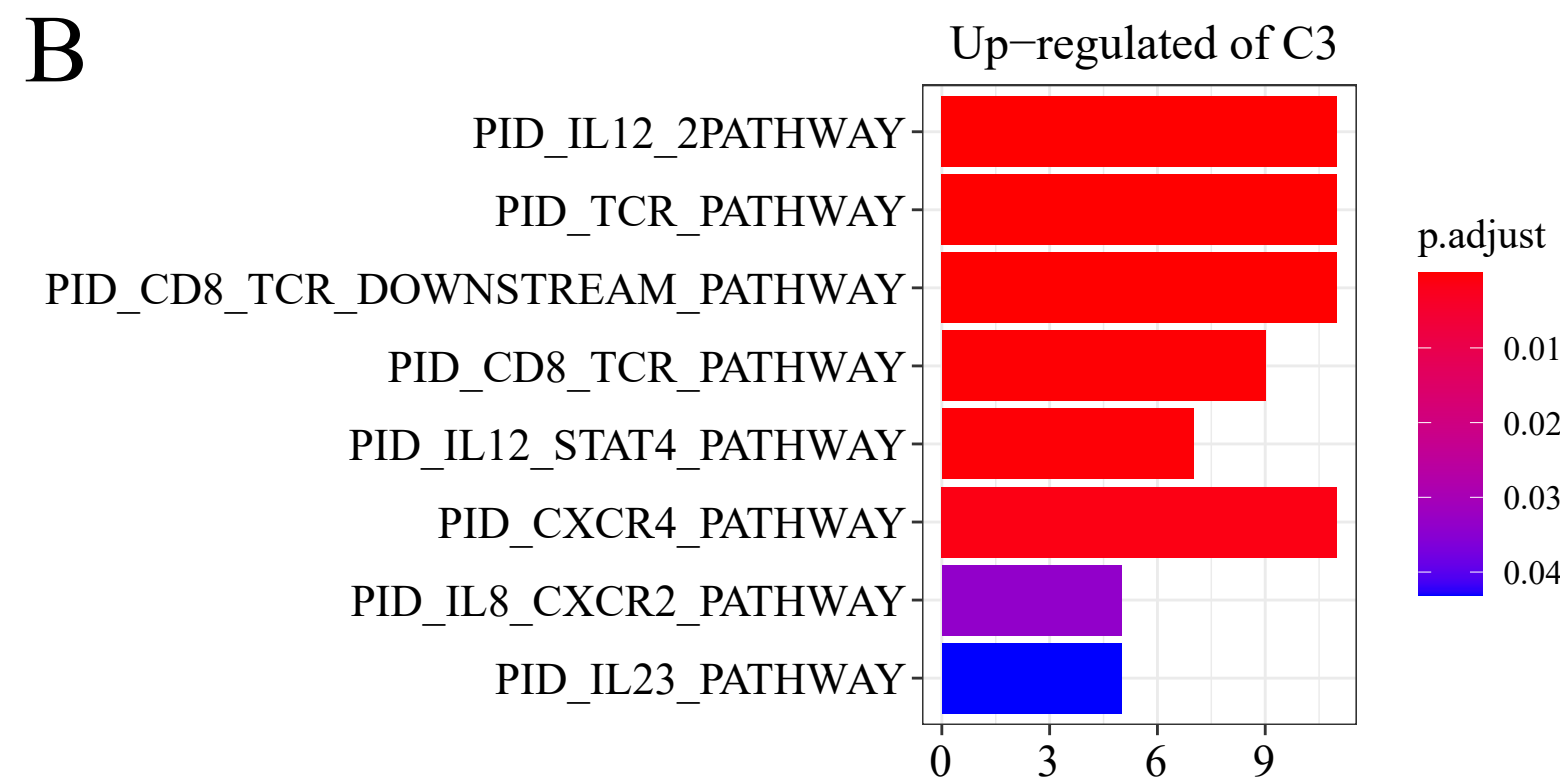

C

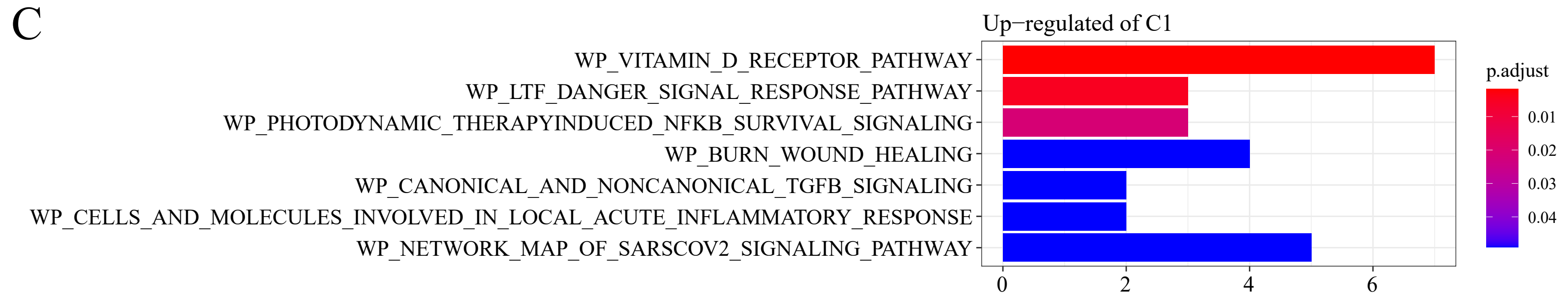

D

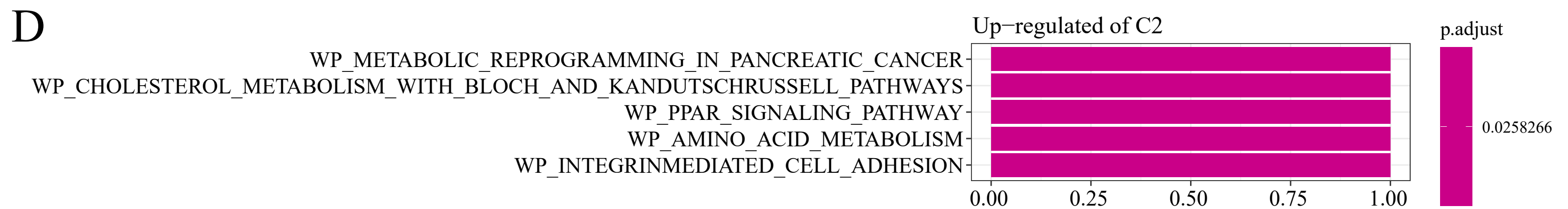

E

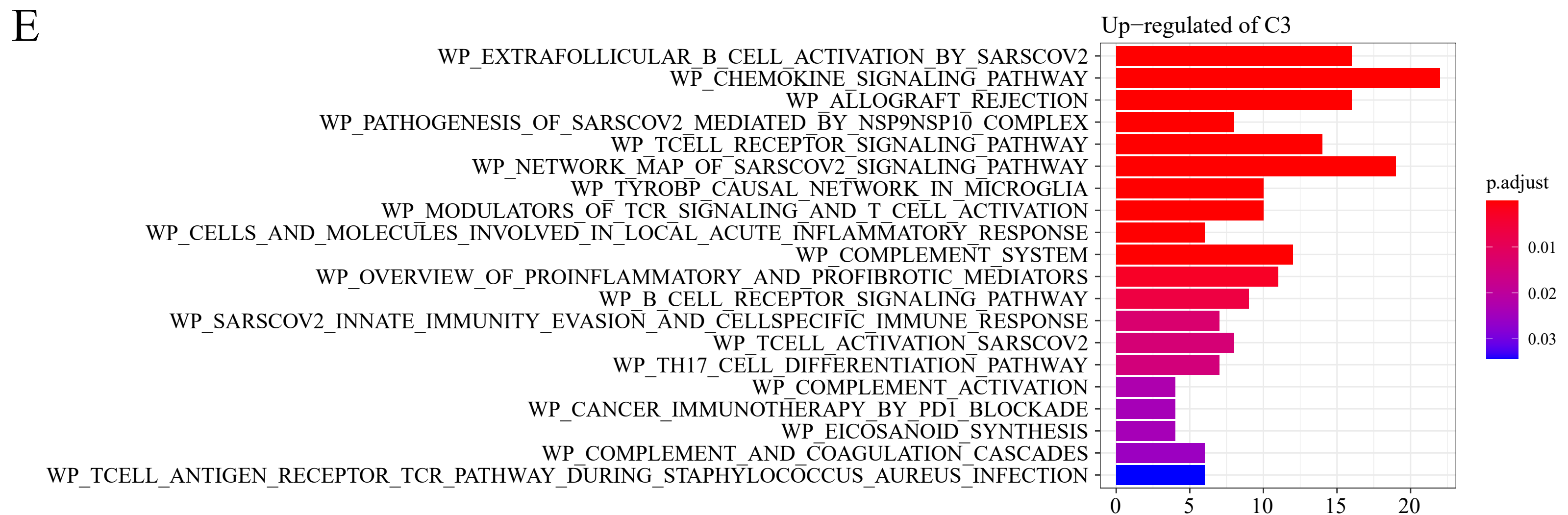

Supplement: Supplementary file 2 [file DataSheet4.PDF]

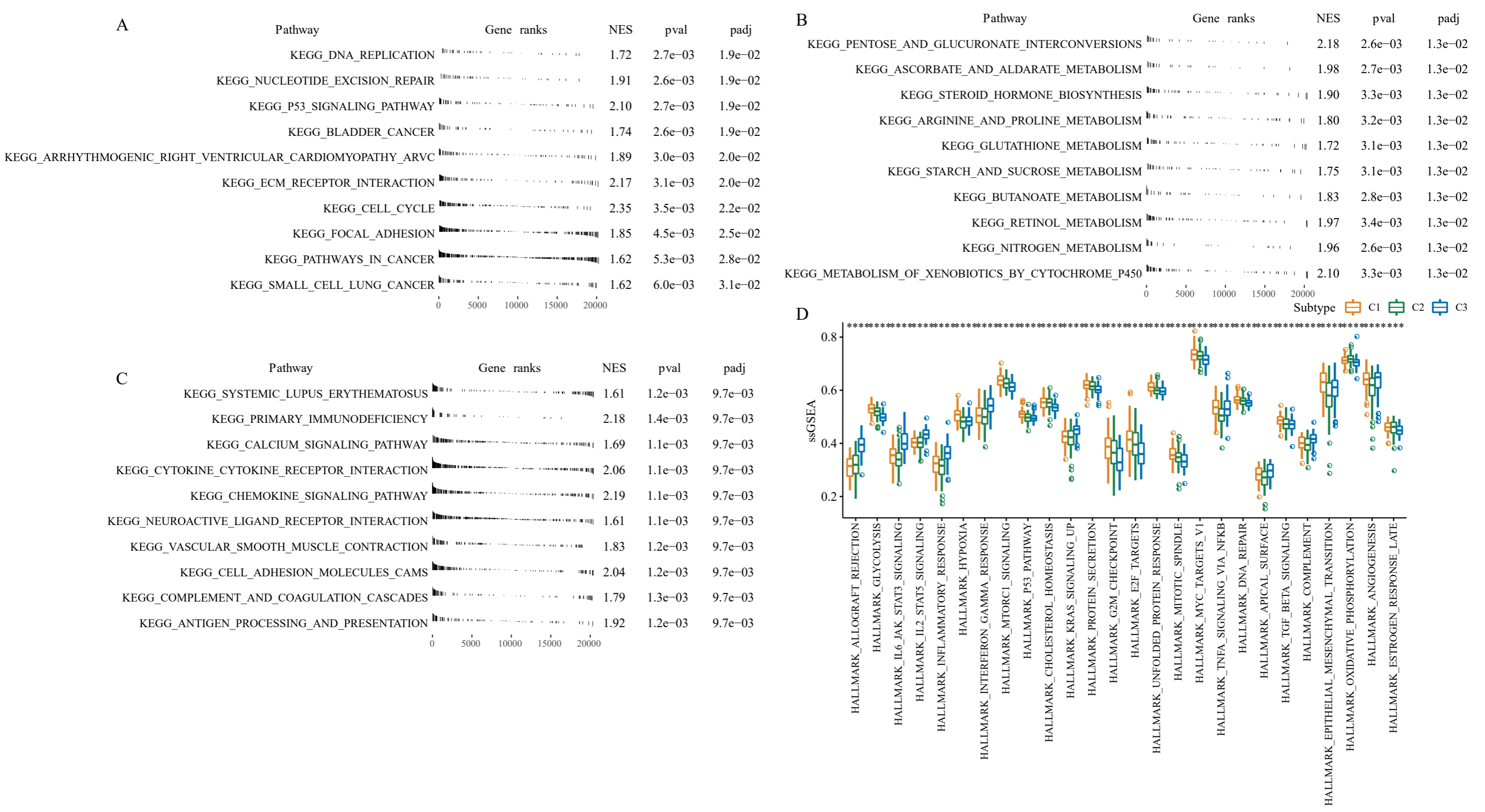

Supplement: Supplementary file 4 [file DataSheet3.PDF]

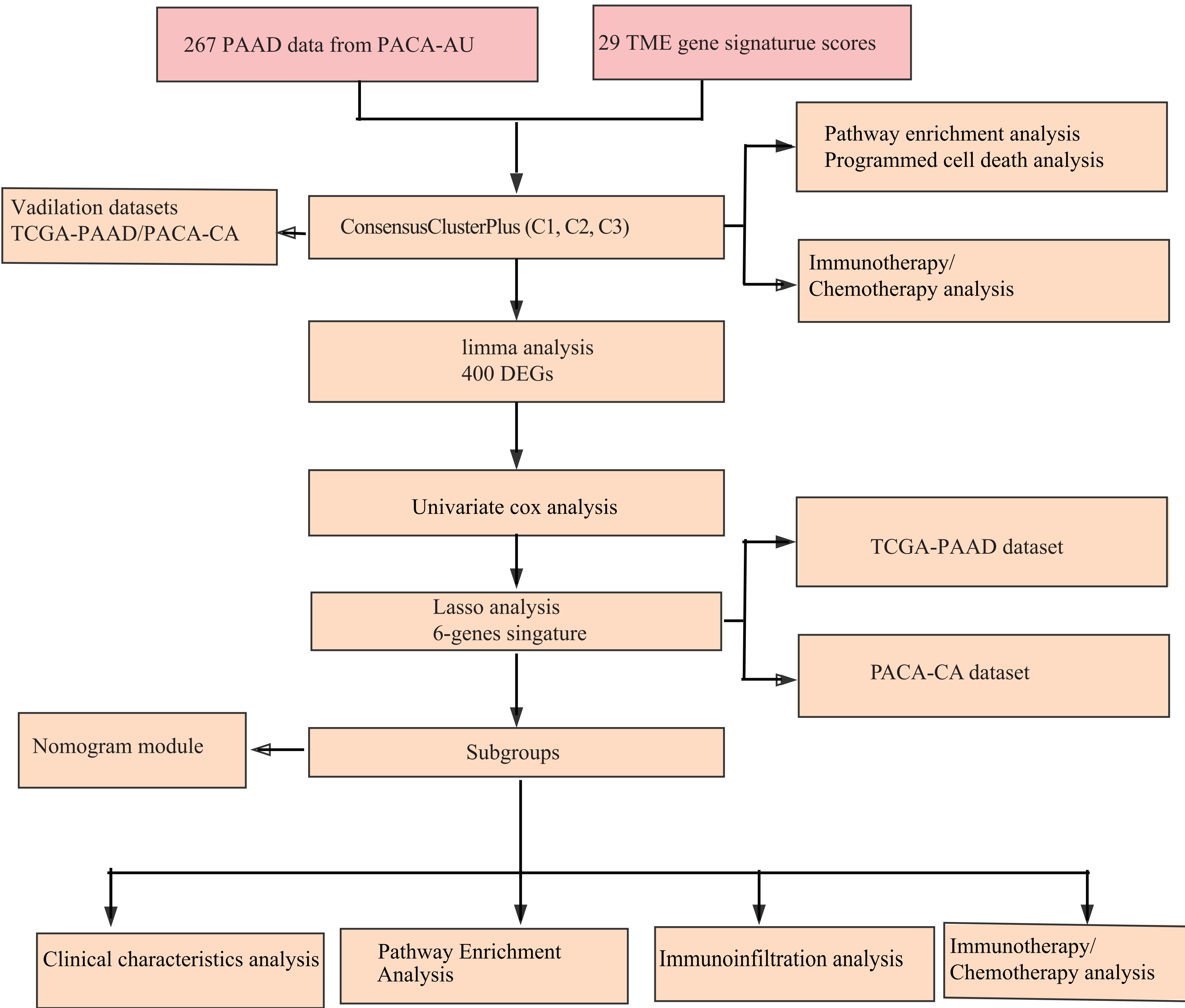

Supplement: Supplementary file 5 [file DataSheet1.PDF]
