# Supplementary material for: Characterization of tumor microenvironment and programmed death-related genes to identify molecular subtypes and drug resistance in pancreatic cancer
Source: Front Pharmacol. 2023 Mar 17;14:1146280. doi: 10.3389/fphar.2023.1146280 (PMC10063807; doi:10.3389/fphar.2023.1146280)
Supplement: Supplementary file 3 [file Table1.docx]

**Supplementary figure legends**

Figure S1 Working flow chart

Figure S2 Identification of TME molecular subtype (A) Cumulative distribution function (CDF) curve (B) CDF Delta area curve, which indicates the relative change in the area under the CDF curve for each category number k compared with k-1. The horizontal axis represents the number k and the vertical axis represents the relative change in the area under the CDF curve (C) The heatmap corresponding to the consensus matrix for k =3 obtained by applying consensus clustering. The rows and columns of the matrix represent samples.

Figure S3 Differences in biological pathways between molecular subtypes (A) The GSEA algorithm was performed with all KEGG gene sets in C1 vs Other in PACA-AU cohort (B) The GSEA algorithm was performed with all KEGG gene sets in C2 vs Other in PACA-AU cohort (C) The GSEA algorithm was performed with all KEGG gene sets in C3 vs Other in PACA-AU cohort (D) Heatmap of pathway enrichment score between molecular subtypes. *** p<0.001; **** p<0.0001.

Figure S4 Pathway Interaction Database (PID) and Wiki Pathways (WP) analysis of up-regulated genes in C1, C2 and C3 groups in the PACA-AU cohort.
